# Metagenomic analysis of ancient dental calculus reveals unexplored diversity of oral archaeal *Methanobrevibacter*

**DOI:** 10.1186/s40168-021-01132-8

**Published:** 2021-09-30

**Authors:** Lena Granehäll, Kun D. Huang, Adrian Tett, Paolo Manghi, Alice Paladin, Niall O’Sullivan, Omar Rota-Stabelli, Nicola Segata, Albert Zink, Frank Maixner

**Affiliations:** 1 Institute for Mummy Studies, Eurac Research, 39100 Bolzano, Italy; 2 Faculty of Biology, Department of Biology II, Anthropology and Human Genomics, Ludwig-Maximilians-University of Munich, 82152 Planegg-Martinsried, Germany; 3 CIBIO Department, University of Trento, 38123 Trento, Italy; 4 Department of Sustainable Agro-Ecosystems and Bioresources, Fondazione Edmund Mach, 38010 San Michele all’Adige, Italy; 5 CUBE - Division of Computational Systems Biology, Centre for Microbiology and Environmental Systems Science, University of Vienna, Vienna, Austria; 6 Center Agriculture Food Environment, University of Trento, 38123 Trento, Italy

**Keywords:** Ancient DNA, Ancient dental calculus, Oral microbiome, Metagenomics, De novo assembly, *Methanobrevibacter*

## Abstract

**Background:**

Dental calculus (mineralised dental plaque) preserves many types of microfossils and biomolecules, including microbial and host DNA, and ancient calculus are thus an important source of information regarding our ancestral human oral microbiome. In this study, we taxonomically characterised the dental calculus microbiome from 20 ancient human skeletal remains originating from Trentino-South Tyrol, Italy, dating from the Neolithic (6000–3500 BCE) to the Early Middle Ages (400–1000 CE).

**Results:**

We found a high abundance of the archaeal genus *Methanobrevibacter* in the calculus. However, only a fraction of the sequences showed high similarity to *Methanobrevibacter oralis*, the only described *Methanobrevibacter* species in the human oral microbiome so far. To further investigate the diversity of this genus, we used de novo metagenome assembly to reconstruct 11 *Methanobrevibacter* genomes from the ancient calculus samples. Besides the presence of *M. oralis* in one of the samples, our phylogenetic analysis revealed two hitherto uncharacterised and unnamed oral *Methanobrevibacter* species that are prevalent in ancient calculus samples sampled from a broad range of geographical locations and time periods.

**Conclusions:**

We have shown the potential of using de novo metagenomic assembly on ancient samples to explore microbial diversity and evolution. Our study suggests that there has been a possible shift in the human oral microbiome member *Methanobrevibacter* over the last millennia.

Video abstract

**Supplementary Information:**

The online version contains supplementary material available at 10.1186/s40168-021-01132-8.

## Background

Dental calculus develops via the mineralisation of plaque, which can remain for millennia on ancient skeletal remains. It displays a specific microniche in the oral cavity and has been shown to perfectly preserve ancient biomolecules (DNA, proteins, metabolites) [1–3] and dietary microfossils (pollen, starch) [4]. Ancient dental calculus has therefore been used as a source of information to study the composition and functional properties of oral microbial communities, diets and health in the past [5, 6]. For example, changes in the oral microbial composition from the Neolithic to the Industrial Revolution have indicated that dietary shifts have played a key role in altering our oral microbial ecosystems [7]. Further studies of ancient calculus have identified the presence of various keystone oral pathogens [5], in particular members of the so-called red-complex, a group of bacteria highly associated with periodontal disease [8], as well as the presence of inflammatory host response proteins [5, 9–14].

Despite this, little is known about the role and diversity of non-bacterial microbes in ancient dental calculus. In comparison to modern plaque and calculus, ancient calculus has reportedly a higher abundance of archaea, dominated by the genus *Methanobrevibacter* [13]. Currently, *Methanobrevibacter oralis* is the only isolated and characterised *Methanobrevibacter* species in the human oral microbiome [15], but undetermined *Methanobrevibacter* species have been continuously found in ancient dental calculus [6, 11, 14, 16] in as high abundances as >60% [14, 17]. This frequent occurrence has allowed the reconstruction of a draft genome of a member of *Methanobrevibacter* from a Neanderthal calculus which was denominated *Methanobrevibacter oralis neanderthalensis*, the only reconstructed ancient *Methanobrevibacter* genome to date [6]. Despite the high abundance of *Methanobrevibacter* spp. in ancient dental calculus and its possible involvement in periodontal disease [18, 19], the current knowledge on the diversity and role of these methanogens in the human oral microbiome is limited.

**Fig. 1:**
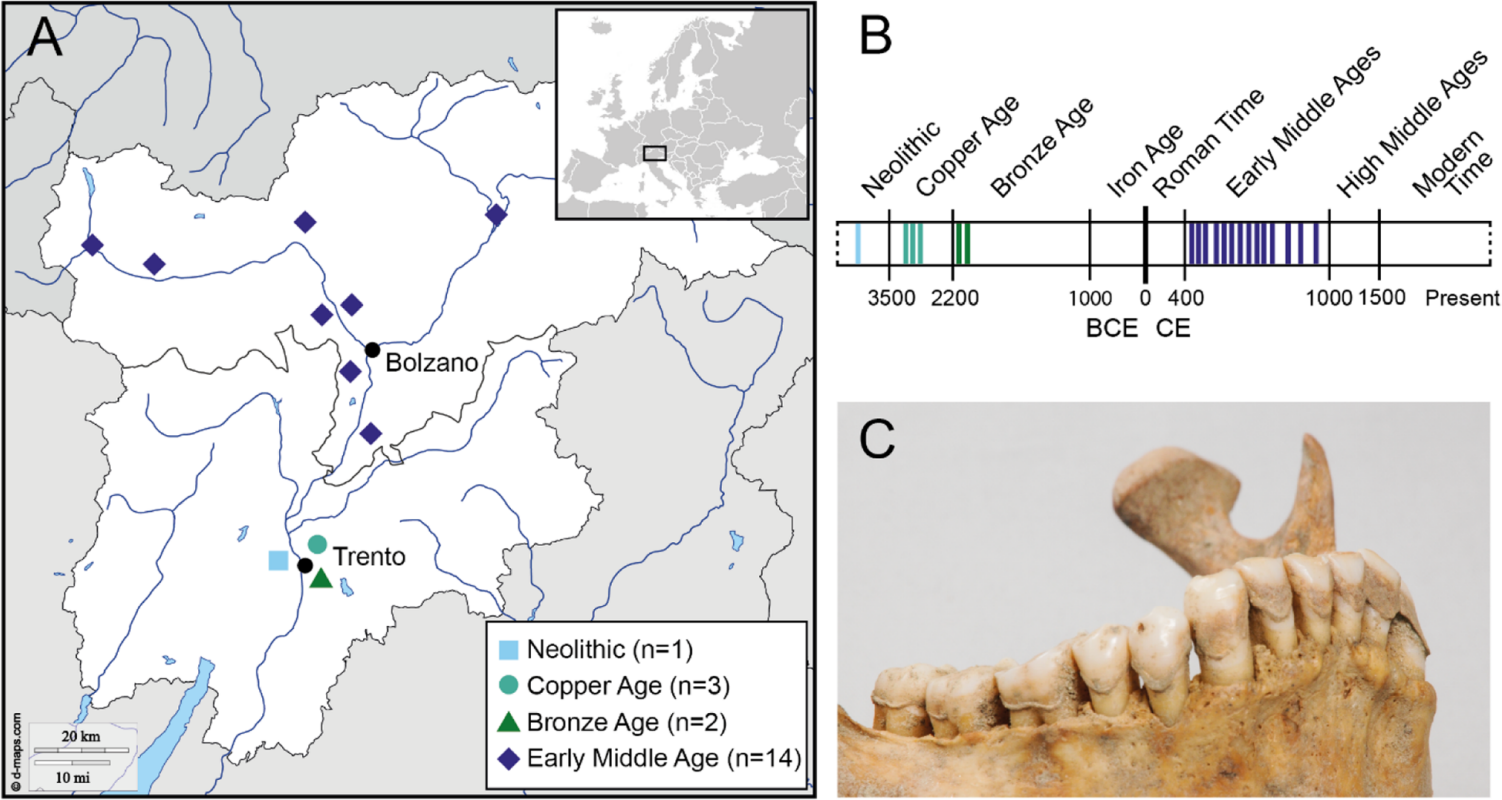
**A** Spatial and **B** temporal location of the 20 dental calculus samples in this study. Archaeological time periods based on [20]. **C** Dental calculus on the mandibular teeth of the early medieval individual 2100, found in the burial site of Burgusio Santo Stefano (St. Stephan ob Burgeis) in South Tyrol, Italy

To better understand the complex diversity and evolution of the oral microbiome, here we analysed dental calculus from ancient human remains from Trentino-South Tyrol region in Northern Italy, spanning from the Neolithic to the Early Middle Ages (Fig. 1). Apart from studying the overall microbial community of the samples, we focused on the less studied archaeal component of the oral microbiome and de novo metagenomic assembled ancient genomes of members of the genus *Methanobrevibacter*. We discovered two hitherto unknown archaeal *Methanobrevibacter* species and show that the actual *Methanobrevibacter* genetic diversity is much larger than previously thought. This has important implications for understanding the evolution of the dental calculus microbiome in humans in the last few millennia.

## Methods

### Individuals

Ancient dental calculus from 20 individuals found in various archaeological sites in Trentino-South Tyrol, Italy, spanning from the Neolithic to the Early Middle Ages [20] (Fig. 1, Additional file 2: Table S1), were subjected to in-depth metagenomic analysis to characterise the oral microbiome and further describe the diversity of the genus *Methanobrevibacter*. Anthropological analysis (sex and age at death estimations) and radiocarbon dating of the human remains from the Early Middle Age were previously published in [21], and the prehistoric individuals were analysed according to the same methods. Periodontitis was evaluated to be present or absent, where it was present if the distance between the cemento-enamel junction (CEJ) and the margin of the alveolus was more than 3 mm (modified from [22]) (Additional file 2: Table S1). Severity of caries was determined using a degree scale scoring system of 0–4, where 0=no carious lesion detected, 1=beginning of dentin demineralisation, 2=dentin demineralisation without pulp exposure, 3=dentin demineralisation with pulp exposure and 4=large carious lesion, tooth retained, with pulp exposure [23].

### Sampling, DNA extraction and sequencing

All genetic laboratory work was conducted in the dedicated ancient DNA lab of the Institute for Mummy Studies at Eurac Research, Bolzano, Italy. Dental calculus (10–66mg) was removed from the surface of each tooth using sterile tweezers and probes. Each sample was sprayed with 3% H_2_O_2_ and then exposed to UV light for 10 min to sterilise the calculus surface.

Overnight DNA extraction was done using a solution of 0.5M EDTA, 20 mg/ml proteinase K and 0.1M N-Laurylsarcosine, modified from [24, 25]. Extracts were concentrated using 10K Amicon® filter devices and purified using Qiagen® MinElute PCR Purification Kit. Extraction blanks were included for every five samples. Single-indexed adapted libraries were prepared according to Meyer and Kircher 2010 [26] with unique 7 bp indexes. The libraries were pooled and paired-end sequenced on an Illumina Hiseq4000, including one of the extraction blanks. Data is available from the European Nucleotide Archive under accession no. PRJEB43389.

### Pre-processing sequencing data

Paired Illumina sequences were merged and adapters were trimmed using PEAR [27] with a minimum merging overlap and minimum sequence length of 25 bp. Per base quality was set to 25, and trimmed using QualityFilterFastQ [28]. After quality control, all samples were filtered against the reads in the extraction blank to reduce cross contamination effects from index hopping [29], using bbduk from the BBtools package (https://sourceforge.net/projects/bbmap/) [30].

### Pre-processing comparative datasets

For comparative analysis, previously published ancient and modern dental calculus metagenomic sequences from humans, Neanderthals and baboons [5, 6, 11, 13, 31] were downloaded and pre-processed as described above. Cutadapt [32] was used to remove adapters from single end read dataset, with otherwise the same quality cut-offs. Modern plaque, tongue dorsum, stool and skin samples were collected from the human microbiome project (HMP) [33] (Additional file 2: Table S2). Soil samples were downloaded from a study of Johnston and colleagues [34]. Modern tongue dorsum, skin, stool, plaque and soil datasets were trimmed and adapters removed with Cutadapt, with a minimum sequence length of 30 bp, and the per base quality cut off of 20. Only samples reported with low contamination and with >6,000,000 reads after pre-processing were used for comparative and statistical analysis to match the amount of reads in the Trentino-South Tyrolean calculus dataset.

### Authentication

After pre-processing, taxonomic assignment was performed using MetaPhlAn2 (version 2.7.7) [35]. For ancient samples, the non-default minimum read length threshold was set to 30 bp to adjust for short aDNA fragments (--read_min_len 30).

To authenticate the metagenomic sequences as coming from an ancient oral source, we first used Sourcetracker2 [36] on species-level data to identify the source of possible contaminants. Other comparative datasets were used as sources (soil, tongue dorsum, skin and modern calculus).

A species-level PCoA was also performed to confirm the oral origin of the ancient dental calculus sequences in comparison to microbial communities from plaque, tongue dorsum, skin, modern and ancient calculus. Bray-Curtis distances were calculated based on the normalised taxonomic assignment from MetaPhlan2 using Qiime2 [37] and visualised with ggplot2 (https://ggplot2.tidyverse.org/) [38].

To assess typical ancient DNA damage of the metagenomic reads, MapDamage2 [39] was run on sequences aligned to the genomes of microorganisms detected in the calculus samples. Pre-processed reads were aligned to the highest abundant identified taxa (> 2.3% average abundance) in the Trentino-South Tyrolean calculus dataset, as well as the members of the red complex: *Bacteroidetes* oral taxon 274 (NZ_GG774889.1), *Desulfobulbus* oral taxon 041 (GCA_000349345.1), *Eubacterium saphenum* (NZ_GG688422.1), *Fretibacterium fastidiosum* (GCA_000210715.1), *Methanobrevibacter oralis* DSM 7256 (GCF_001639275.1), *Porphyromonas gingivalis* (NC_010729.1), *Pseudopropionibacterium propionicum* (NC_018142.1), *Streptococcus sanguinis* (NC_009009.1), *Tannerella forsythia* (NC_016610.1) and *Treponema denticola* (NC_002967.9). Alignment to microbial genomes was performed using Bowtie2 [40], with the ‘very sensitive local setting’ (-D 20 -R 3 -N 1 -L 20 -I S,1,0.50) and deduplicated using DeDup v0.11.3 (https://github.com/apeltzer/DeDup). Alignment quality was set to 30.

### Human DNA analysis

Pre-processed sequences from the ancient dental calculus were aligned to the human reference genome (build hg19) using BWA [41] with seed disabled and then deduplicated using DeDup. Minimum mapping quality was set to 30. MapDamage2 was used to assess ancient DNA damage on the human reads. Genetic sexing was performed as described in [42]. Genetically determined sex was used for subsequent analysis, except when the genetic sex could not be determined in which case anthropologically determined sex was used.

### Calculus microbiome taxonomic characterisation

Prior to the diversity and statistical analysis, all samples were normalised to 6,000,000 reads (using seqtk version 1.3-r106, https://github.com/lh3/seqtk) to match the lowest number of sequences in the Trentino-South Tyrolean dataset. Bray-Curtis distances between oral microbiomes (ancient calculus, modern calculus, plaque, tongue dorsum) were calculated based on the normalised taxonomic assignment from MetaPhlan2 using Qiime2 [37] and visualised with ggplot2.

### Analysis of *Methanobrevibacter* abundance

To determine differences in microbial composition between ancient and modern calculus, especially differences in *Methanobrevibacter* abundance, a linear discriminant analysis was performed on phylum, genus and species abundances using LEfSe [43]. The threshold on the logarithmic LDA score for discriminative features was set to 4. Differences in phylum abundance between ancient and modern was visualised using pheatmap in R [44], only showing phyla >2% abundance. A t-test from the R package ggpubr [45] was used to determine differences in each phylum that were statistically significant between the two groups.

We tested whether there was any correlation between the abundance of *Methanobrevibacter* and various categories of metadata (level of periodontitis, age, sex, time period, etc.) using Kruskal-Wallis rank sum test and pairwise Wilcoxon tests in R. Correlations between species were calculated with Spearman’s correlation with the Hmisc package in R [46].

### Microbial genome reconstruction from ancient calculus samples

Each of the 20 pre-processed ancient calculus samples in this study was subjected to de novo metagenome assembly using metaSpades (version 3.10.1; default parameters) which was evaluated to outperform among other metagenome assemblers [47, 48]. We obtained 92,485 contigs (> 1000 nt) which were kept for further processing. Reads were aligned against contigs using Bowtie2 (version 2.2.9; ‘--very-sensitive-local’) and the output was used for contigs binning using MetaBAT2 (version 2.2.9; ‘-m 1500’), resulting in 117 bins (i.e. putative genomes) [40, 49]. The putative genomes were assessed for completeness, contamination and strain heterogeneity using CheckM (version 1.0.7; lineage specific workflow), to select the set of final draft genomes considered [50]. Based on recent guidelines, we selected medium-quality (MQ) genomes that had completeness > 50% and contamination < 5%, resulting in 76 metagenome assembled genomes (MAGs) [30]. Each of the microbial genomes was assessed for genome size (bp), number of contigs, contig N50 values, mean contig length and the longest contig (Table S7) using QUAST [51].

To assert the endogenous origin of reconstructed microbial genomes, we checked each genome for nucleotide misincorporation rate patterns which instrument to authenticate ancient sequences. The BAM files of alignment between reads and contigs were processed using mapDamage2 (default parameters) [39]. Finally, we assigned a taxonomic label to each reconstructed microbial genome using PhyloPhlAn3.0 (phylophlan_metagenomic, ‘-d SGB.Aug19’) [52].

### Phylogenetic analysis of *Methanobrevibacter* genomes

To place the newly reconstructed ancient *Methanobrevibacter* genomes in the phylogenetic context of the *Methanobrevibacter* genus, we used a total of 64 modern assembled genomes (52 reference genomes (Additional file 2: Table S13) and 12 MAGs from a previous large-scale metagenomic investigation [53]) representative of 17 known *Methanobrevibacter* species. Core genes were searched within ancient MAGs (n = 11) and their contemporary counterparts (n = 64) and were then concatenated into a core gene alignment of 42,225 bp length using a Roary pipeline (version 3.13.0; ‘-i 80 -cd 90 -e -mafft’) [54]. To reconstruct the phylogenetic tree, we used RAxML (8.1.15) [55] under a GTR model of substitution with 4 gamma categories and 100 bootstrap pseudo replicates.

We then sought to reconstruct a more precise phylogeny for three subtrees partitioned from the *Methanobrevibacter* genus tree built as described above, using two similar but complementary methods. Firstly, we performed the phylogenetic analysis using RAxML (as described above) on the core gene alignment using genomes from three subtrees, respectively. Core gene alignments were produced using PRANK [56] with parameters of 85% identity (90% for genomes in the subtree 3 due to the fact that subtree 3 contains fewer genomes) for gene clustering and of gene presence in >90% across genomes for defining core genes for each subtree. Secondly, we used the same RAxML phylogenetic method on the multiple sequence alignment (MSA) reconstructed based on the whole genome region. The whole-genome MSA for three subtrees were reconstructed, by firstly aligning whole genome sequences (as query genomes) from each subtree against one selected reference genome (GCA_003111605 for subtree 1; GCA_003111625 for subtree 2; GCA_001639275 for subtree 3) with BLASTn (‘-word_size 9’) [57]. Afterwards, genomic regions where query genomes and the reference genome share a substantial sequence similarity (BLASTn hits with length >500 bp and identity percentage >95%) were selected to generate the whole-genome MSA, followed by excluding columns having >10% missing data.

To further support the phylogenetic distances with a method based on the genome similarity, we also estimated the average nucleotide identity (ANI) pairwise distances using pyani (version 0.2.6; option ‘-m ANIm’) [58] for two subtrees (subtree 1 and subtree 2) which comprise the 10 newly reconstructed and uncharacterised *Methanobrevibacter* genomes from our ancient calculus samples. The measurement was performed on the whole genome sequences and on the core gene alignments generated above as well.

Next, we explored the strain-level phylogenetic diversity of the three *Methanobrevibacter* species with particular interest (the two newly discovered* Methanobrevibacter* species and *Methanobrevibacter oralis*) including 82 publicly available oral microbiome samples (from 10 contemporary and 72 ancient individuals) (Additional file 2: Table S2). An alignment-based approach was used to reconstruct the whole genome alignment. GCA_001639275 was selected as a reference for a known species, *M. oralis*, and reconstructed MAGs with highest completeness and lowest contamination were selected as representatives of ancient lineages from subtree 1 and those from subtree 2 (here calc_2086.bin.1 for those from subtree 1 and calc_2094.bin.7 for those from subtree 2) due to a lack of reported reference in the public database (Additional file 2: Table S7). For previously published metagenomic samples, draft genomes were generated using a python script consensus.py from package cmseq (https://github.com/SegataLab/cmseq). It aligned metagenomic reads of each sample against single references and extracting consensus sites of aligned reads. Sites covered by reads were filled with gaps if: (1) mapping quality < 30, (2) coverage < 3 folds, (3) minimum identity of reads < 97%, (4) aligned read length < 30nt, and (5) minimum dominant allele frequency < 80%. Newly sequenced samples of this study from which *Methanobrevibacter* genomes could not be obtained by de novo assembly were also subjected to the same approach. For other newly sequenced samples whose MAGs are available, we aligned contigs against the same selected references using BLASTn (‘-word_size 9’). A whole-genome MSA was compiled based on the same single reference, integrating reconstructed draft genomes and highly-similar sequences (>95% identity percentage and >500 bp length) of aligned MAGs. We cleaned each alignment, excluding sequences with >50% gaps and then removing columns containing >10% missing data. The cleaned alignments were used in reconstructing the phylogenetic trees with RAxML as above. Recombination events for these two subtrees were analysed using ClonalFrameML (v1.25, default parameters) under a ML phylogenetic context. Alignments were masked from recombination by replacing genomic regions affected by recombination with gaps using python script maskrc-svg (https://github.com/kwongj/maskrc-svg). The ML phylogeny was estimated for masked alignments. The process was iteratively repeated until no recombination events were detected. To confirm the phylogeny built on the MAGs from this study, we also reconstructed draft genomes from short-sequencing reads of the same metagenomic samples using the alignment-based approach as described above and then repeated the same phylogenetic analysis.

### Functional annotation and pangenome analysis of the ancient *Methanobrevibacter* bins

In order to analyse the potential difference in function between the three *Methanobrevibacter* species (*M. oralis*, TS-1 and TS-2), the assembled ancient *Methanobrevibacter* bins were annotated with prokka [59] and UniRef90 [60]. The pangenome for each of the three species was determined using Roary [54] as described above. The *M. oralis* pangenome was created using the four *M. oralis* assembled genomes (GCA_001639275, GCF_000529525.1, GCF_900289035.1, and GCF_902384065.1) present in Genbank, as well as the ancient *M. oralis* bin from sample 2102. We firstly determined known functional features that were unique to the two ancient *Methanobrevibacter* pangenomes by excluding those found in the *M. oralis* pangenome. To curate the automatic selection for functional features, we then manually inspected the gene tables, removed *M. oralis* orthologs found in OrthoDB (vs 10.1) and finally conducted a blastn search to find any similar sequences in *M. oralis* and other *Methanobrevibacter* species.

To further study the gene content differentiation, we analysed gene absence and presence across these three species based on sequence clustering. The pangenome across TS-1, TS-2 and *M. oralis* was generated as described above and the predicted genes were annotated with UniRef90 and eggNOG 5.0 [61] using eggNOG-mapper v2 [62] (with default settings). We focused only on genes which were shared by 90% of the genomes from one species but completely absent in the other two species in order to differentiate the functional and metabolic potentials across these three species.

Lastly, we screened each pangenome of TS-1, TS-2 and *M. oralis* for the presence of antimicrobial resistance genes and carbohydrate-active enzymes which are related to modern human lifestyles. We compared predicted genes and the respective genomes to a Comprehensive Antibiotic Resistance Database (CARD) using RGI [63]. A further validation was performed by using another approach, abricate (https://github.com/tseemann/abricate), which covers a larger database [64–71]. For assessing carbohydrate-active enzymes, each pangenome was searched using HMMER (E-value < 1e−15 as cutoff) [72] in dbCAN2 (http://bcb.unl.edu/dbCAN2/blast.php), an automated CAZyme annotation platform.

### Methyl-coenzyme M reductase (*mcrA*) gene analysis

The methyl-coenzyme M reductase complex (Mcr) is a key enzyme in methanogenesis, and one of its subunits, *mcrA*, is a common marker gene for methanogenic archaea [73]. To determine if this pathway was present in the ancient genomes, we extracted the *mcrA* gene sequences from the *Methanobrevibacter*-assembled genomes after annotation with prokka. For samples that had not generated any *Methanobrevibacter* bins the metagenomic sequences were aligned to the *mcrA* gene from their phylogenetically closest genomes (*M. oralis* DSM 7256, TS-1 or TS-2). These additional *mcrA* sequences were extracted from the aligned bam-files with ANGSD (version 0.918) and samtools faidx (version 1.10) [74, 75]. Only bases covered at least 3 times and with a minimum alignment score of 30 were used for the consensus sequence (angsd -dofasta 2 -doCounts 1 -setMinDepth 3 -minQ 30). The *mcrA* dataset was complemented with *mcrA* sequences from currently available genomic datasets of species in the genus *Methanobrevibacter* (n=13). First, all DNA sequences were translated into amino acids by using the Perform Translation tool in the ARB software package [76]. The *mcrA* sequence alignment was automatically inferred with the ClustalW protein alignment program [77], implemented in the ARB software package and then manually refined by using the ARB sequence editor. The amino acid alignment of selected samples was further examined for the conservation of important positions in the catalytic site [78, 79]. For the phylogenetic analysis of the *mcrA* gene, the nucleic acid sequences were re-aligned according to the corresponding amino acid sequences with the respective tool in the ARB software. Phylogenetic analysis was performed by applying distance-matrix, maximum-parsimony, and maximum-likelihood methods implemented in the ARB software package: neighbour-joining (using the Jukes-Cantor algorithm for nucleic acid correction with 1000 bootstrap iterations), DNA parsimony (PHYLIP version 3.66 with 100 bootstrap iterations), and DNA maximum-likelihood [PhyML [80] with the HKY substitution model]. In total, 1541 alignment columns were used for phylogenetic analysis.

## Results

We sampled the dental calculus from 20 individuals found in 11 burial sites located in Trentino-South Tyrol (Fig. 1, Additional file 2: Table S1), Italy, and subjected them to shotgun metagenomic sequencing. Most of the individuals showed signs of oral diseases, with 80% (16/20) of them affected by periodontitis. Seventy-five percent (15/20) of the individuals showed carious lesions of very high level (destructive decays) (Additional file 2: Table 2). Only two individuals (10%) did not show any signs of oral disease.

### Taxonomic characterisation and authentication of the ancient calculus microbiome

**Fig. 2:**
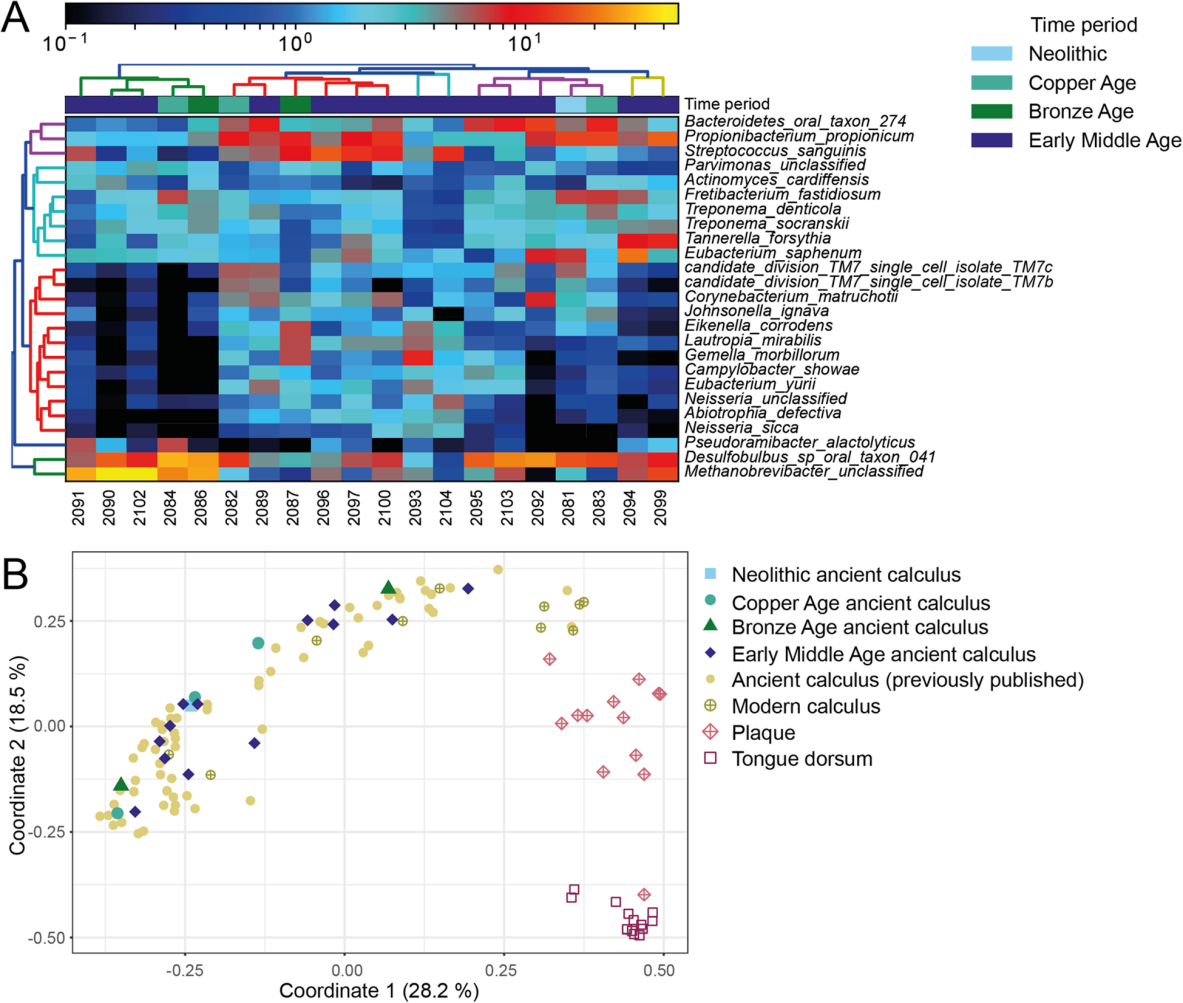
Taxonomic analysis of the microbial composition of the ancient dental calculus samples collected. **A** MetaPhlAn heatmap of the top 25 species found in the ancient calculus. **B **Species-level principal coordinate analysis (PCoA) of β-diversity (Bray-Curtis distances) considering the microbiome from ancient calculus (this study) [5, 6, 11, 13, 31] modern calculus [13], plaque and tongue dorsum (HMP) [33]

To authenticate the sequences as coming from an oral source and to determine the composition of the microbiome, they were first taxonomically profiled with MetaPhlAn 2 (Fig. 2A). We found a total of 222 microbial species in the Trentino-South Tyrolean dataset (Additional file 2: Table 3). The most abundant phyla were Firmicutes, Proteobacteria, Actinobacteria, and Bacteroidetes, consistent with the reported major phyla in the expanded Human Oral Microbiome Database eHOMD and other ancient calculus microbiomes [5, 6, 81]. Of the 25 highest abundant species (Fig. 2A), 21 were categorised as oral in eHOMD, and 13 of them have been associated with periodontitis. These include the ‘red complex’ members *Treponema denticola* and *Tannerella forsythia* [8], but also species from the genera *Methanobrevibacter* and *Desulfobulbus*, anaerobes found in periodontal deep pockets and regarded as ‘late colonisers’ of oral plaque [18, 19]. Further species-level PCoA analysis based on Bray-Curtis distances showed that our dental calculus samples cluster together with previously published ancient calculus samples (Fig. 2B) and showed similarities to modern calculus (mineralised plaque sampled from living humans, as opposed to the living biofilm which constitutes plaque), but were distinctly different from the other modern human datasets used for comparison (plaque, tongue dorsum, skin and stool) (Additional file 1: Figure S1). We did not find any further clustering of the calculus samples based on the time period of the sample or the health status, origin, sex and age at death of the individuals (Additional file 1: Figure S2). Additional analysis using Sourcetracker2 also showed that of the reads stemming from a known source, a majority were from oral sources (predominantly modern calculus and plaque) (Additional file 1: Figure S3).

All samples displayed post-mortem damage typical for ancient DNA for the selected highest abundant microbial species (Additional file 1: Figure S4, Additional file 2: Table S4), indicating that the reads are stemming from the ancient calculus and not from recent microorganisms. In addition to the microbial DNA, the calculus metagenomic sequences contained on average 0.12% human DNA (Additional file 2: Table S5). Genetic sex of the human host could be determinated for 18 of the individuals. Post-mortem damage in the human reads was detected in all samples (Additional file 2: Table S5). Altogether, all samples were considered authentic and used for downstream analysis, except sample 2100 which had substantially fewer reads and was therefore not used for further comparative analysis.

### *Methanobrevibacter* is highly abundant in ancient calculus

**Fig. 3:**
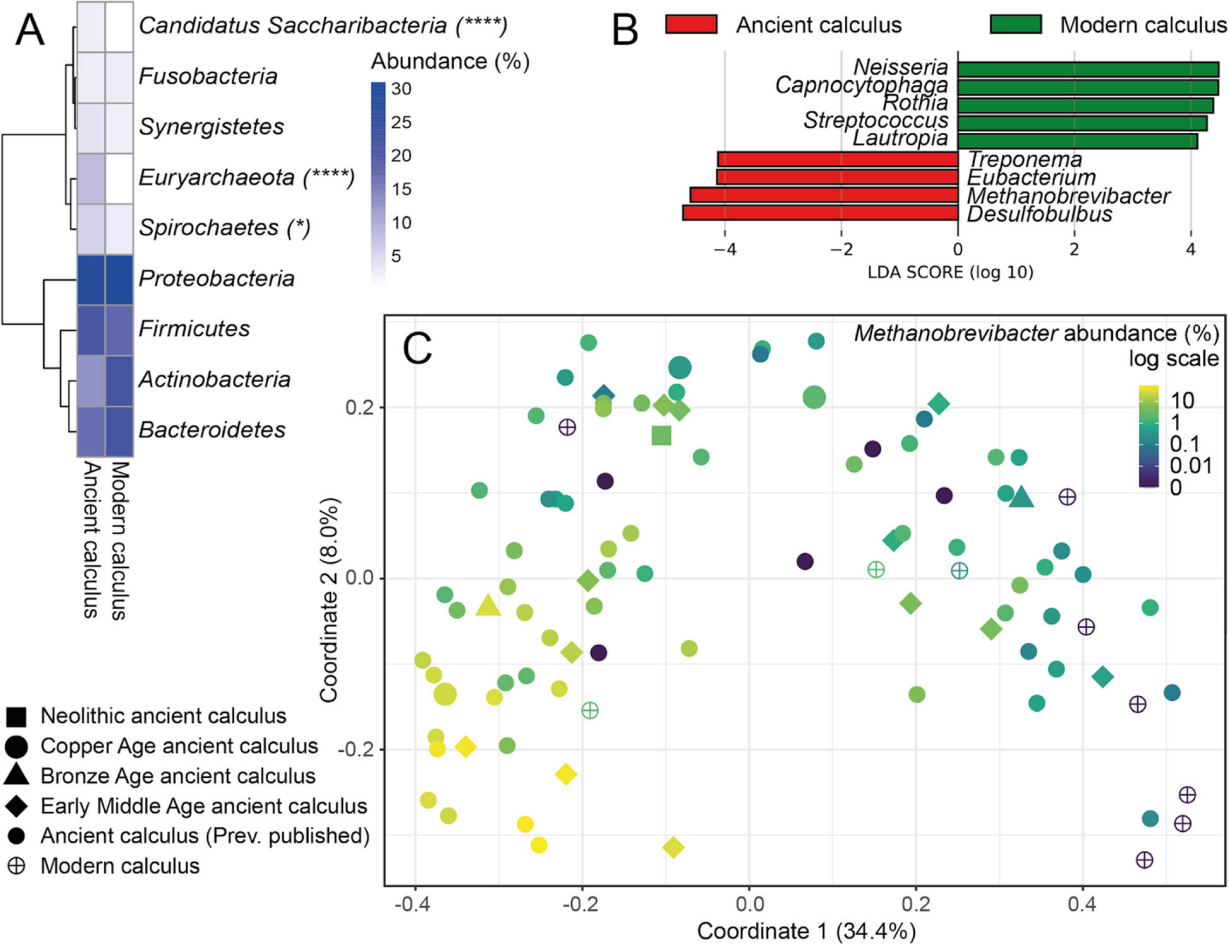
Differences in microbial taxonomic profiles between ancient and modern calculus and *Methanobrevibacter* abundance in dental calculus. **A** Phylum abundance (%) in ancient and modern calculus. Phyla that are statistically different between the two tissues are marked with asterisks (*p ≤ 0.05, ****p ≤ 0.0001). **B** Differences in genera between ancient and modern calculus (LEfSe) [43]. **C** Species-level principal coordinate analysis of the β-diversity (Bray-Curtis distances) of all ancient and modern calculus samples showing the abundance of *Methanobrevibacter*. Previously published ancient calculus from [5, 6, 11, 13, 31] and modern calculus from [13] were used in the analysis

To determine if there was a difference in microbial community composition between modern and ancient calculus, we compared the taxonomic profiles of our samples to a total number of 76 published ancient and modern calculus samples (Additional file 2: Table S2). We observed in both ancient and modern calculus microbiomes high abundances of members of Firmicutes, Proteobacteria, Actinobacteria and Bacteroidetes, whereas members of Euryarchaeota, Fusobacteria, Synergistetes, Spricohaetes and Candidatus Saccharibacteria were underrepresented, indicating a similar pattern between the ancient calculus and its contemporary counterpart regarding the taxonomic composition at the phylum level (Fig. 3A). However, ancient calculus had on average 8 times higher amounts of Euryarchaeota than modern calculus (p=2.4e-07) (Fig. 3A). This was represented by the genus *Methanobrevibacter*, the only archaeal genus present in the calculus samples which was found to be one of the major features to explain the differences between ancient and modern calculus microbiomes (LDA score = 4.62) (Fig. 3B). Other species significantly represented in the ancient calculus include *T. denticola*, *E. saphenum* and *Desulfobulbus* oral sp. 041 (Additional file 1: Figure S5, Additional file 2: Table S6).

*Methanobrevibacter* sequences were present in all Trentino-South Tyrolean samples (0.11–47.3% relative abundance), and in five samples it constituted the dominant taxa of the whole microbiome (22.5–47.3% relative abundance) (Additional file 2: Table S3). The abundance of *Methanobrevibacter* was one of the driving components of the clustering of calculus microbiomes, displaying a gradient of abundance across the first coordinate of the PCoA plot which attributed to 34.4% of the variance in the dataset (Fig. 3C). Statistical analysis revealed no correlations between the abundance of *Methanobrevibacter* and different groups of metadata in all calculus samples, including the recorded oral diseases, sex and age at death. Looking at correlations between abundances between *Methanobrevibacter* and other species, we found several positive and negative correlations (Additional file 2: Table S6). Two out of four observed positive correlations of *Methanobrevibacter* was to other bacterial species that have been associated with periodontitis, e.g. *Filifactor alocis* (corr=0.3328, p<0.001), and *Streptococcus anginosus* (corr=0.3377, p<0.001).

### Metagenome assembly of calculus samples reveals two novel *Methanobrevibacter* species, TS-1 and TS-2

We sought to reconstruct bacterial and archaeal genomes from ancient calculus specimens from the Italian Trentino-South Tyrolean region, performing de novo metagenome assembly on each calculus metagenomic sample. From the 20 calculus, we reconstructed a total of 117 metagenome assembled genomes (MAGs). All reconstructed MAGs were processed by strict quality measurement including completeness, contamination and strain heterogeneity, resulting in 76 of them to be considered of medium quality (MQ) or above as proposed by recent guidelines [30] (completeness > 50% and contamination < 5%). The endogenous origin of these 76 genomes was authenticated with each displaying clear damage patterns at the both ends of reads (Additional file 1: Figure S6). At the species level, only 16 of the genomes are close enough (Mash distance [82] < 5%) to the genome with a known species-level taxonomic label as defined by the Species-level Genome Bin (SGB) analysis [52, 53] (Additional file 2: Table S8). The remaining genomes (n=60) were assigned as unknown species.

**Fig. 4:**
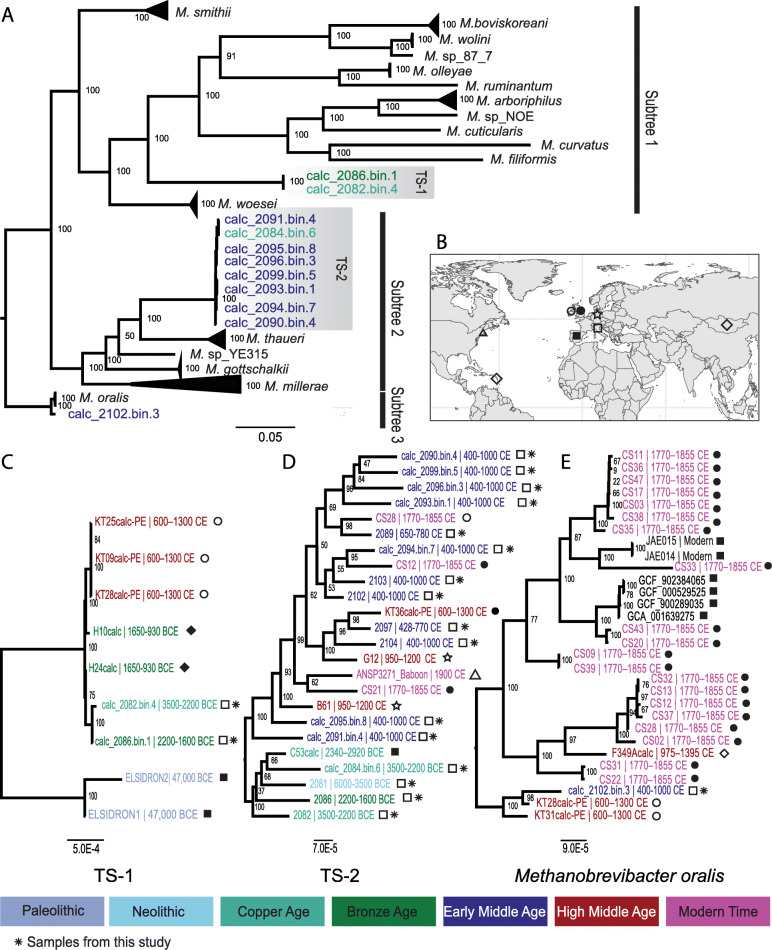
Phylogenetic analysis of ancient and modern *Methanobrevibacter* diversity. The colours in the trees corresponds to the age of the samples: Paleolithic: >12,000 BCE, Neolithic: 6000–3500 BCE, Copper Age: 3500–2200 BCE, Bronze Age: 2200–1000 BCE, Early Middle Age: 400–1000 CE, High Middle Age: 1000–1300 CE, Modern time: 1492 CE–present. **A** Phylogenetic tree showing the *Methanobrevibacter* MAGs with modern *Methanobrevibacter* genomes. **B** Geographical location of ancient calculus samples placed in the phylogenetic context in (C–E). **C** Phylogenetic tree of ancient calculus samples aligned to the highest quality TS-1 MAG (>50% covered at least 3 folds). **D** Phylogenetic tree of ancient calculus samples aligned to the highest quality TS-2 MAG (>50% covered at least 3 folds). **E** Phylogenetic tree of samples aligned to *M. oralis* (50% covered at least 3-folds)

Additionally, a total of 11 ancient genomes were identified to be within the *Methanobrevibacter* genus (Additional file 2: Table S8) and fell in three separated subtrees when placed in phylogenetic context with all publicly available representatives of the genus *Methanobrevibacter* (Fig. 4A). One genome fell within the *M. oralis* clade with a Mash distance < 2% from the *M. oralis *reference genome, and the other 10 ancient genomes formed two independent clades which were clearly distinct from other *Methanobrevibacter* species. The phylogeny of each subtree was independently reconstructed using the maximum likelihood method which resulted in an improved branch length resolution (Additional file 1: Figure S7). The ANI pairwise distances between the newly reconstructed *Methanobrevibacter* genomes and the closest modern relatives showed a limited distance among ancient genomes based on both the core genome and whole genome (Additional file 1: Figure S8). Conversely, ancient genomes were strikingly distant from the modern closest relatives, with mean distance >15% in subtree 1 and subtree 2, in terms of both core and whole genome (Additional file 1: Figure S8). The high ANI distance between ancient genomes and modern representatives, in both subtree 1 and subtree 2, suggests that the ancient genomes could represent two newly discovered species which have not yet been previously reported based on current operational ANI-based consensus on assigning strains to species [53, 83–86].

The distinction of ancient genome clades in subtree 1 and subtree 2 is further supported by the clade separation with a clearly long branch length in the subtree phylogeny based on core genome as well as based on whole genome (Additional file 1: Figure S7A, S7B, S7C and S7D). These two candidate species (TS-1 for ancient genomes from subtree 1 and TS-2 from subtree 2) thus represent novel lineages that have not previously been found in modern calculus metagenomic datasets so far.

### Phylogenomic analysis provides historical insights into temporal diversity of three ancient-sample enriched *Methanobrevibacter* species

Studies focusing on modern oral microbiomes have consensus on the pivotal role methanogenic archaea play in human oral health [19, 87, 88]. The medium-quality *Methanobrevibacter* genomes we recovered from the ancient calculus samples of this study allowed us to study their evolutionary diversity, during a long-term period, at an unprecedented resolution. We firstly explored the phylogenetic position of newly reconstructed *Methanobrevibacter* MAGs in the whole diversity of genus *Methanobrevibacter *(Fig. 4A). This was further supported by the consistent phylogenetic structure reconstructed independently using genomes from each subtree (Additional file 1: Figure S7).

Secondly, to study the biodiversity of three species represented by the 11 *Methanobrevibacter* MAGs obtained in this study, we performed comparative analysis exploiting 82 publicly available calculus metagenomes (Additional file 2: Table S2). Reconstructed *Methanobrevibacter* MAGs were divided into three groups based on their phylogenetic placement: group 1 included genomes from subtree 1, group 2 comprised genomes placed in the subtree 2 and the single genome clustering with *M. oralis* lineages in the subtree 3 was assigned to group 3 (Fig. 4A). We detected genomic signals of those three clades in the publicly available metagenomic calculus samples by aligning metagenomic reads against the selected representative genomes. Specifically, seven samples showed at least 50% of the TS-1 representative genome covered at >3X, 12 samples for TS-2 and 25 samples for *M. oralis* (Additional file 1: Figure S9). These high-coverage samples were further included in the phylogenetic analysis along with *Methanobrevibacter* MAGs reconstructed in this study.

Leveraging a total of 102 calculus samples (20 of this study and 82 from previous studies) which spanned ~50,000 years and were collected from eight countries across three continents shed light on the temporal and geographical diversity, from prehistory to modern era, of three *Methanobrevibacter* species enriched in ancient samples. We used a maximum likelihood approach to build a strain-resolved phylogeny based on the MSA reconstructed aligning metagenomic reads (or MAGs if available in the samples) against genome sequences calc_2086.bin.1, calc_2094.bin.7, and GCA_001639275, respectively representative of TS-1, TS-2, and *M. oralis*. To maximise the number of samples possibly being included without losing phylogenetic resolution, we used only samples which have >50% reference genome covered at >3X and retained only positions covered across >90% of genomes. Additionally, to ameliorate the phylogenetic reliability, we iteratively removed the regions which were affected by homologous recombination until no recombination events were detected. We observed that the novel candidate species TS-1 has been continuously present from Late Pleistocene Age, Copper Age, Bronze Age, Iron Age, to Late Middle Ages (Fig. 4C). The earliest evidence of its presence could be found in two 48,000-year-old Neanderthal samples in one of which a *Methanobrevibacter oralis*-like genome was previously reported to be present as well [6]. The two Neanderthal lineages of TS-1 seem to be the ancestral to the more recent lineages from Italy, Mongolia and Ireland (Fig. 4C). We did not observe any signature of this species in the modern calculus samples, and the latest record could be only traced in medieval samples from Ireland (Fig. 4C, Additional file 1: Figure 9). Likewise, TS-2 has dispersed over multiple countries across continents during at least the last 5000 years (Fig. 4D). Compared to TS-1 and TS-2, *M. oralis*-like strains appear to be more prevalent in relatively recent samples from the sixth century CE to the modern era with a wide genetic diversity across multiple geographical locations (Fig. 4E). Taken together, our phylogenetic analysis indicates that the three *Methanobrevibacter* (*M. oralis*, TS-1, TS-2) found in ancient dental calculus from a broad geographic range show first signs of a temporal diversification. To further verify these findings, it is however necessary to increase the statistical power by including more calculus samples in the future, especially modern samples.

To verify the phylogeny which was reconstructed using MAGs, we also performed the same phylogenetic analysis using draft genomes reconstructed by mapping metagenomic reads against a single reference genome. We obtained identical phylogenies for TS-1 and *M. oralis* (Additional file 1: Figure S10) when metagenomic reads were used directly. This added analysis further supports that the MAGs generated in this study possess sufficiently high sequence quality to enable high resolution phylogenetic analysis. While a certain extent of phylogeny variability was observed in the TS-2 between using MAGs and using draft genomes reconstructed by the alignment-approach, most of these tips had arisen from nodes supported by low bootstrapping values (Additional file 1: Figure S10). The alternated phylogeny observed in TS-2 could also be because of effects from homologous recombination. Our recombination analysis using ClonalFrameML [89] showed that TS-2 had 3 times the amount of recombination events than *M. oralis*, and 30 times that of TS-1 (Additional file 1: Figure S11).

### Functional analysis of the calculus *Methanobrevibacter* species reveal similarities to other *Methanobrevibacter* species

#### Methyl-coenzyme M reductase *mcrA* gene in oral *Methanobrevibacter* species

To characterise functionality in the three calculus *Methanobrevibacter* species, we extended our analysis to one component of the Methyl Coenzyme M Reductase, the key enzyme in methanogenesis which catalyses the final and rate-limiting step in methane biogenesis [73]. We focused our analysis to the *mcrA* subunit encoding gene, a widely-used marker gene for methanogen classification. Importantly, all ancient *Methanobrevibacter* MAGs in the newly described clades harbour the *mcrA* gene. The *mcrA* gene phylogeny of all calculus samples and selected modern *Methanobrevibacter* isolates shows a highly similar tree topology to the whole genome phylogeny and also separates the two new clades from *M. oralis* and other known *Methanobrevibacter* species. (Additional file 1: Figure S12). Further comparison of the *mcrA* amino acid alignment revealed in all three oral *Methanobrevibacter* clades a high conservation of most amino acid residues that were previously described to interact with CoM, CoB, F430 cofactors and that are part of the substrate cavity wall or have post-translational modifications [78, 79]. Only in one catalytic site, which is part of the substrate cavity wall, TS-1 encodes a tyrosine and not a phenylalanine, which both *M. oralis* and TS-2 share at that position (Additional file 1: Figure S13).

#### Species-specific functional genes

To determine functional differences between the two newly discovered *Methanobrevibacter* species present in the ancient calculus samples compared to *M. oralis*, we characterised and annotated their respective pangenomes. 1628 genes were found in the TS-1 pangenome, 2676 genes in the TS-2 pangenome, compared to the 2649 genes in the *M. oralis* pangenome (Additional file 2: Tables S9-S10). Due to the characteristics of ancient DNA (e.g. damage and loss of DNA over time), we only compared genes with known predicted functions that were present in TS-1 and TS-2, but not present in *M. oralis*. Based on UniRef90 annotations we found only four genes in TS-1 identified with different functional features in comparison to *M. oralis*: 2 versions of DNA (Cytosine-5)-methyltransferase 1, which is involved in amino acid metabolism; T/G mismatch-specific endonuclease, involved in DNA repair; and LlaMI, a restriction endonuclease gene (Additional file 2: Tables S11). Further comparative sequence analysis shows that all four genes are also present in *Methanobrevibacter* species other than *M. oralis*, e.g. *Methanobrevibacter smithii* and *Methanobrevibacter millerae*. Likewise, nine genes from TS-2 were identified with different functions which *M. oralis* lacks. The majority are involved in metabolic or transporter pathways. 55.5% (5/9) of the genes showed a high sequence similarity to other *Methanobrevibacter* species, mostly *M. smithii* and *Methanobrevibacter* sp. YE315. Two genes involved in metal uptake indicate a functional differentiation of TS-2 to *M. oralis*. These are the Molybdate ABC transporter permease protein ModB present in 7 of the 8 TS-2 MAGs and the Nicotianamine synthase-like protein present in all TS-2 MAGs.

To further investigate the functional and metabolic diversity across TS-1, TS-2 and *M. oralis*, we characterised the pangenome across these three species using the UniRef [60] and eggNOG databases [61]. Focusing on genes that can be characterised with KEGG orthology, we identified 227 genes that are present in all TS-1 genomes but absent in the other two species. For TS-2 and *M. oralis*, we also found 129 and 98 such species-specific genes, respectively (Additional file 1: Figure S14, Additional file 2: Tables S14-S16). From these species-specific genes, we next sought to identify the ones that also had a unique KEGG orthology (KO) and their respective metabolic pathways. We found 11 unique KOs in TS-1 and 10 in TS-2, and they are mostly involved in basic metabolisms (e.g. carbohydrate, energy and lipid metabolisms), cellular and genetic information processing (Additional file 2: Table S17). Of note, we found only one KO unique to *M. oralis* and it could be related to glycan biosynthesis and metabolism or cationic antimicrobial peptide (CAMP) resistance (Additional file 2: Table S17).

To determine potential adaptations to a higher human intake of complex carbohydrates after the advent of farming and a greater use of antibiotic substances in the modern human lifestyle [90, 91], we then assessed TS-1, TS-2 and *M. oralis* for the presence of antimicrobial resistance genes and CAZyme families. As a result, we could not detect genetic traces related to antimicrobial resistance in the genomes. On the other hand, we found a total of 10 CAZy families in the gene pool of TS-1, TS-2 and *M. oralis*, six of which are common to these three species (Additional file 1: Figure S15). These family members are involved in either assembling or breaking down complex carbohydrates (e.g. cellulose and chitin) (Additional file 2: Table S18).

## Discussion

In this study, we analysed ancient calculus samples utilising de novo metagenome assembly techniques to increase the knowledge of the oral microbiome and its diversity over time, with a particular focus on the periodontitis-associated archaeal genus *Methanobrevibacter*. All analysed samples, independently of the site and the time period, contained both ancient endogenous microbial DNA and traces of the human host DNA. Taxonomic characterisation and authentication of the metagenomic data showed that the studied samples mainly consist of oral microbial members and that there are only few signs of external contamination from other environments (e.g. soil). Overall, the calculus samples of all analysed individuals (independently of the sex, age at death or diseases) had a taxonomic composition similar to what has been previously found in other ancient calculus studies [6, 11, 13] and a major proportion of the discovered microorganisms have been associated with periodontitis [8, 92–95]. These include the red complex members *Treponema denticola* and *Tannerella forsythia* [8] but also species from the genera *Methanobrevibacter* and *Desulfobulbus*, late colonisers of oral plaque and found in deep periodontal pockets [18, 19]. Many of these taxa were also defining for the ancient calculus microbiome, compared to modern calculus samples which were instead characterised by taxa associated with healthy plaque, e.g. *Streptococcus sanguinis*, *Lautropia mirabilis*, *Rothia dentocariosa*, *Neisseria sicca* and Neisseria elongate [96, 97]. This may indicate a difference in the level of maturation, and, possibly, disease state, in the calculus samples, between ancient and modern calculus as previously reported [13], or various taphonomic processes [14].

Consistent with previous observations [11, 13, 14], we also found that *Methanobrevibacter* members are profoundly abundant in our samples (up to 47.3%), which could be related to the association of *Methanobrevibacter* enrichment and oral disease. In modern patients, the abundance of *Methanobrevibacter* spp. have been correlated to the severity of periodontitis (up to 18.5% in severe periodontitis), and *M. oralis* and *Candidatus Methanobrevibacter* sp. has been found in plaque from periodontitis patients as well [19, 98, 99]. However, we could not find any such statistically significant correlation between *Methanobrevibacter* abundance and periodontitis in our dataset. It is important to highlight that 75% of the samples showed signs of periodontitis, which may have biased our correlation analysis. While the correlation was still unclear even when we expanded our dataset by including more previously published ancient and modern calculus samples with reported health status, there were indications of the presence of a non-healthy microbiome in the samples and *Methanobrevibacter* was positively correlated to other species associated with periodontitis (Additional file 2: Table S6). It is thus clear that further studies are needed to shed light on the complexity of the non-healthy oral microbiome and its implications for human health. The role of *Methanobrevibacter* members in human health will be characterised further in the future with an increasing availability of metagenomic samples from ancient and contemporary oral environments.

Of note, the high abundance of *Methanobrevibacter* observed in ancient calculus could also be related to taphonomic processes [14]. Post-mortem decay could skew the abundance of microorganisms in ancient calculus through overgrowth of certain taxa after the individual has died. Similarly, DNA preservation in ancient samples could be biased between microbial species due to their genomic GC-content [11] or differences in cell wall composition [5, 100] which could both confer stability to DNA after death. Furthermore, as calculus has been created over the lifetime of an individual, the abundance of taxa in ancient calculus most likely does not represent a single snapshot of a microbial community. The plaque microbiome can also change over time in one individual [101], which makes quantitative comparisons between plaque, modern and ancient calculus difficult.

The classical alignment-based methods depending on reference genomes are common in ancient DNA studies [6, 102, 103], but they hamper the detection of new species, genome rearrangement and large indels or regions which are no longer present in extant microbial genomes. Abundant archaeal reads in the calculus samples allowed us to perform de novo metagenomic assembly, which resulted in the reconstruction of 11 novel ancient *Methanobrevibacter* genomes. Unexpectedly, besides only one genome clustering with a known species, *M. oralis*, the other 10 ancient genomes formed two independent clades (TS-1 and TS-2) which are clearly distinct to other *Methanobrevibacter *species (Fig. 4A). The ancient genomes within each new clade were all closely related to each other and highly dissimilar from modern genomes within their respective subtree (>15% ANI) (Additional file 1: Figure S8). This suggests the presence of a higher diversity of human oral *Methanobrevibacter* than has been previously known and confirms that de novo assembly is a valuable tool for identifying new microbial species [104, 105]. Combining ancient and modern metagenomic datasets will therefore support the study of microbial ecology and evolution [106–108]. Many challenges still come with the assembly of ancient DNA due to its fragmented and damaged nature, one limitation being the risk of incorporation of ancient DNA damage into the assemblies. However, we found no evidence of misincorporation in either the damage patterns retrieved after realigning the reads from each sample to their respective MAG (Additional file 1: Figure S6), or the comparison of genomes reconstructed by alignment-based methods or de novo assembly (Additional file 1: Figure S10).

After screening our samples, and the 82 previously published ancient and modern calculus samples, we found sequences from at least one of the three oral *Methanobrevibacter* species (*M. oralis*, TS-1 and TS-2) in 61 samples (59.8%) (Fig. 4C–E). We also found a trend in the prevalence of *Methanobrevibacter* pertaining to sample age. *M. oralis* was more prevalent in modern and younger ancient calculus, and the oldest calculus sample containing >50% coverage of *M. oralis* was the sample 2102 dating back to the seventh century. In contrast, TS-1 was more prevalent in older samples and was completely absent in calculus younger than 700 years. Of the 8 samples containing the highest coverage of TS-1, six were over 3000 years old, including the 48,000-year-old Neanderthal sample. TS-2 was found in both prehistoric and younger samples, spanning at least 6000 years. The phylogenetic analysis of TS-2 also showed that the prehistoric genomes within this species fall together in the tree. We did not observe a clear geographical pattern associating with the phylogeny of the three *Methanobrevibacter* species, but they were found in a broad range of geographical regions in Eurasia and Americas (Fig. 4B–E). As there are only a few publicly available metagenomic datasets from modern calculus samples, it is also possible that TS-1 and TS-2 are more prevalent in modern oral microbiomes than currently known. Sampling more calculus samples and deeper periodontal pockets could help unearth more *Methanobrevibacter* genomes in the future.

The coexistence of different clade members in the same ancient calculus raises the question whether there are any functional differentiations between the clades that could explain the sharing of the same ecological niche. Initially focusing on only genes with known UniRef90 annotations, we observed similar functional features between *M. oralis*, TS-1 and TS-2. For example, all MAGs belonging to TS-1 and TS-2 contained the *mcrA* gene, one of the key genes in methanogenesis, indicating that members of the two new clades use the same anaerobic respiratory pathway like *M. oralis*. However, we also detected two genes present in more than half of the TS-2 genomes that are absent in *M. oralis* and that could potentially confer additional nutritional advantages and niche adaptation within the oral cavity. Several metals, like iron, are a limited resource in the oral cavity [109] and these genes could therefore potentially confer a nutritional advantage to TS-2. One of the genes, present in all eight TS-2 MAGs, is coding for a Nicotianamine synthase protein. Archaeal homologues of this gene have been found in other *Methanobrevibacter* species, e.g. *Methanobrevibacter ruminantium*. Nicotianamine likely also facilitates metal uptake as it displays a high affinity for, and forms complexes with, several metal ions [110]. The other gene enriched in TS-2 is modB, which encodes the Molybdate ABC transporter permease protein ModB, a part of the molybdpterin biosynthesis pathway and methanogenesis [111]. Genes in this pathway have been found to be the result of lateral gene transfer (LTG) in *M. smithii*, potentially as an adaptation to the human gut and increasing metal uptake. It is possible that TS-2 also acquired this transporter via LGT, which could have increased the affinity for, and therefore uptake of molybdate, increasing the methanogenesis. In absence of this transporter, molybdate can be transported via other transporters, e.g. the sulfate/thiosulfate ABC-transporter [112], which is present in *M. oralis*. However, the specific molybdate ABC transporter is more effective at molybdate transport, and the presence of this gene could therefore potentially confer an advantage over other *Methanobrevibacter* that does not carry it.

Our additional gene content analysis instead revealed a higher functional diversity across these three species, identifying 227 genes unique to TS-1, and 129 unique to TS-2. Within these species-specific genes, TS-1 and TS-2 had 11 and 10 unique functional KEGG orthologies, respectively. One of the unique functions found in TS-2 was the gene coding for pyrodixine kinase involved in Vitamin B6 metabolism. This enzyme is a phosphotranspherase which synthesises pyridoxal 5′-phosphate (PLP), the active form of Vitamin B6, needed for more than 140 different metabolic activities in the cell, mostly within amino acid synthesis and degradation [113]. In lieu of this enzyme, we found the PDX1 and PDX2 in *M. oralis*, which is part of an alternative pathway to synthesise PLP and widespread in archaea, plants and fungi [114].

We could not find any genes pertaining to antimicrobial resistance in the ancient or modern *Methanobrevibacter* genomes. Antibiotic-resistance genes are considerably less enriched in ancient coprolites relative to human gut microbiota [115], possibly due to a greater modern day exposure to antibiotics. However, it still remains to be determined if this also holds true for the oral microbiome. Furthermore, we found only a few differences in CAZymes between the ancient and modern *Methanobrevibacter* genomes. Functional validation analyses [116] on different *Methanobrevibacter* isolates might be necessary in the future to determine the role of these genes in conferring advantages in the oral cavity. Due to the low amount of functional features that differ between the three oral *Methanobrevibacter* species, it is difficult to infer with certainty if the possible decline of TS-1 and TS-2 is due to competition by *M. oralis* or to a lack of genetic pathways that could confer an advantage in the modern oral cavity compared to pre-industrialised diets. While we concede it is challenging to draw a complete picture of *Methanobrevibacter* functionality based on a limited number of genomes, the assessment of these three species in the functional and metabolic context set a great proxy for investigating further evolution and adaptation of *Methanobrevibacter* species functionality in the future.

## Conclusions

In this study, we have shown the potential of using de novo metagenomic assembly on ancient DNA sequences to explore the diversity and evolution of oral microbial members. Our analysis unearthed two newly discovered *Methanobrevibacter* species prevalent in calculus from individuals living several thousands of years ago and indicated a possible decline of *Methanobrevibacter* diversity in the human oral microbiome over time. Previous studies have similarly suggested a change in the oral microbiome over time, displaying less diversity observed in modern samples [7]. This is in line with a 16S rRNA gene analysis based on calculus samples dating back to the fourteenth to nineteenth centuries, showing a decreasing *Methanobrevibacter* diversity but with an increase of *M. oralis* in the modern population [16]. However, such decline in the microbiome diversity over time seems not to be specific to the oral environment. Much of the human microbiome diversity today is found in non-Westernised populations [53, 117]. For example, the diversity of a common gut microbe, *Prevotella copri*, has been shown to be higher in modern non-Westernised populations, as well as in ancient individuals, compared to Westernised populations, indicating a shift in diet is a likely factor of the decline in human microbiome diversity [83]. Conversely, some human pathogens have been shown to become more specialised and diverse during the Neolithisation process [103]. Changing lifestyles and diets over the centuries and the modern-day medical usages (e.g. antibiotics) might play a considerable role in depleting human microbiome diversity [7, 90], and hence, it may help explain the observed loss of *Methanobrevibacter* diversity in the recent past. Further studies with fast-growing metagenomic data from both ancient and contemporary populations will certainly enhance our understanding of *Methanobrevibacter* members whose diversity has not been fully unravelled yet.

## Supplementary Information

**Additional file 1: Supplementary Figures S1-S15.**

**Additional file 2: Supplemental Tables S1-S18.**

## Data Availability

The datasets generated and analysed during the current study are available in the European Nucleotide Archive repository under accession no. PRJEB43389. Main scripts used in post-processing and visualising are hosted on GitHub: https://github.com/SegataLab/calculus_study_Lena_2021
