# Supplementary material for: Metagenomic analysis of ancient dental calculus reveals unexplored diversity of oral archaeal Methanobrevibacter
Source: Microbiome. 2021 Sep 30;9:197. doi: 10.1186/s40168-021-01132-8 (PMC8485483; doi:10.1186/s40168-021-01132-8)
Supplement: Supplementary file 2 — Additional file 1: Supplementary Figures S1-S15. [file 40168_2021_1132_MOESM2_ESM.docx]

# **Supplemental figures for “****Metagenomic analysis of ancient dental calculus reveals unexplored diversity of oral archaeal Methanobrevibacter”**

Lena Granehäll^1,2*#^, Kun D. Huang^3,4*^, Adrian Tett^3,5^, Paolo Manghi^3^, Alice Paladin^1^, Niall O’Sullivan^1^, Omar Rota-Stabelli^4,6^, Nicola Segata^3#^, Albert Zink^1^, Frank Maixner^1#^

^1^ Institute for Mummy Studies, Eurac Research, 39100 Bolzano, Italy.

^2^ Faculty of Biology, Department of Biology II, Anthropology and Human Genomics, Ludwig Maximilians University of Munich, 82152 Planegg-Martinsried, Germany

^3^ CIBIO Department, University of Trento, 38123 Trento, Italy

^4^ Department of Sustainable Agro-Ecosystems and Bioresources, Fondazione Edmund Mach, 38010 San Michele all'Adige, Italy.

^5^ CUBE - Division of Computational Systems Biology, Centre for Microbiology and Environmental Systems Science, University of Vienna, 1090 Vienna, Austria.

^6^ Center Agriculture Food Environment, University of Trento, 38123 Trento, Italy


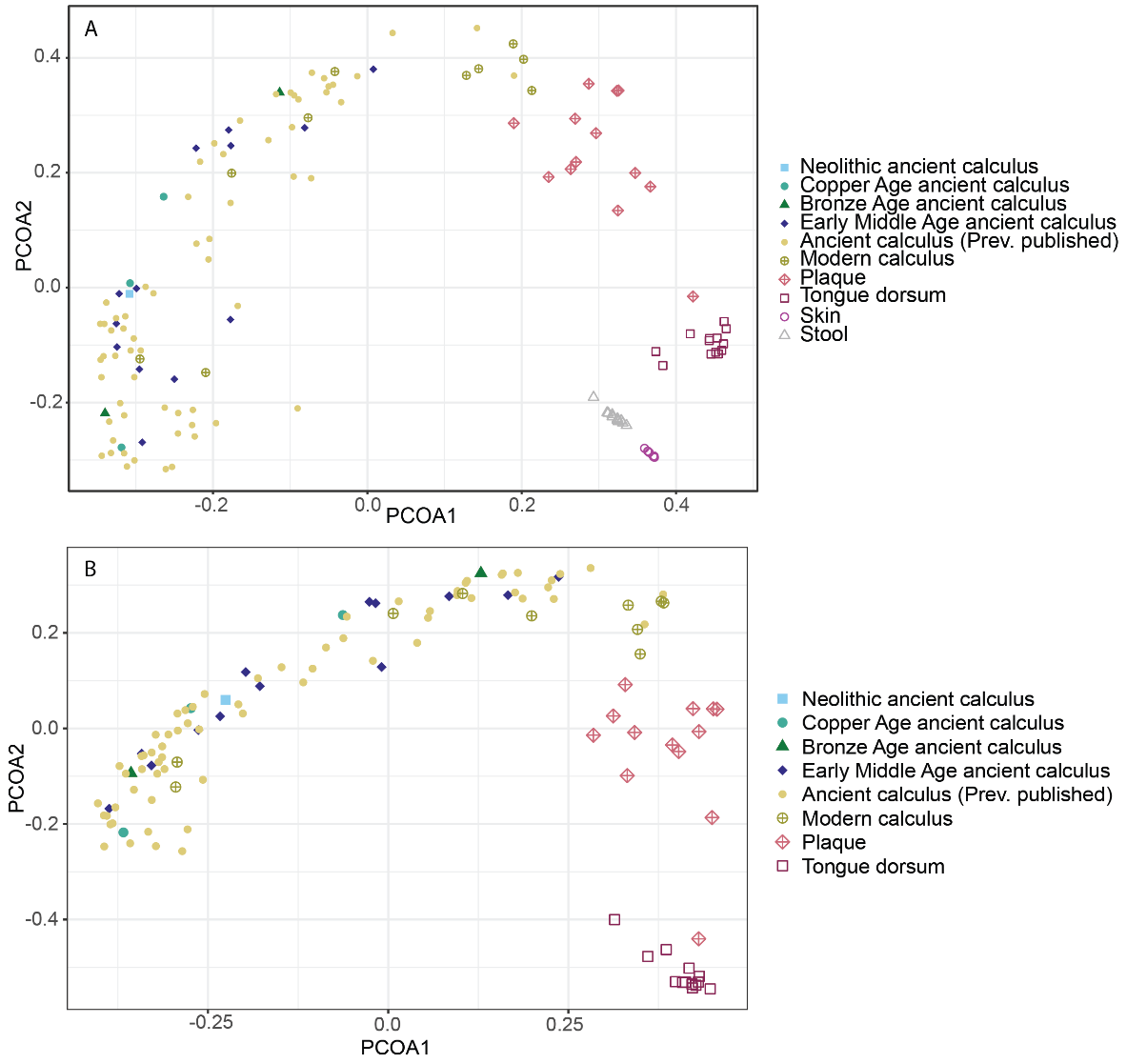


**Figure S1.** A) Species-level principal coordinate analysis of all comparative datasets used in this study (ancient calculus[1-5], modern calculus[1], plaque[6], tongue dorsum[6], skin[6], stool[6] and soil[7]). B) Species-level principal coordinate analysis with oral datasets and filtered to only oral species. The list of 235 oral taxa used for this analysis were determined by identification of taxa present in all oral datasets from the HMP (buccal mucosa, hard palate, keratinized gingiva, palatine tonsils, saliva, subgingival plaque, supragingival plaque, throat, tongue dorsum), where the taxa needed to be present at > 0.1% in at least one oral sample as well as present at any abundance in at least 10% of the total number of oral samples to be included.

**
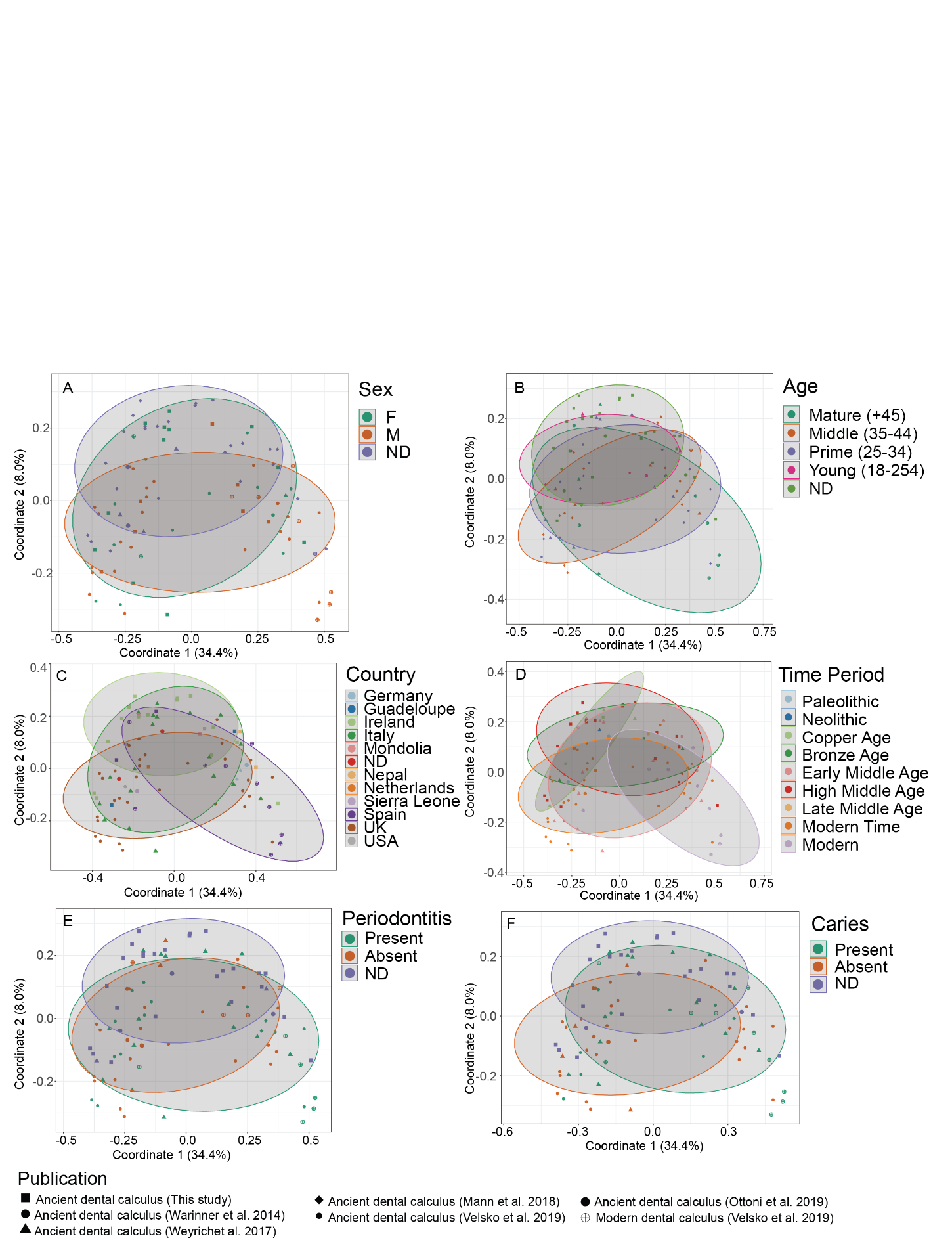
**

**Figure S2.** Species-level Principal coordinate analysis plots based on Bray-curtis distances, visualising normal confidence ellipses (levels=0.75) for each group of metadata: A) Sex B) Age at death C) Country D) Time period of individual (based on archaeological periods from Festi et al. 2014[8]). E) Occurrence of periodontitis. F) Occurrence of caries.


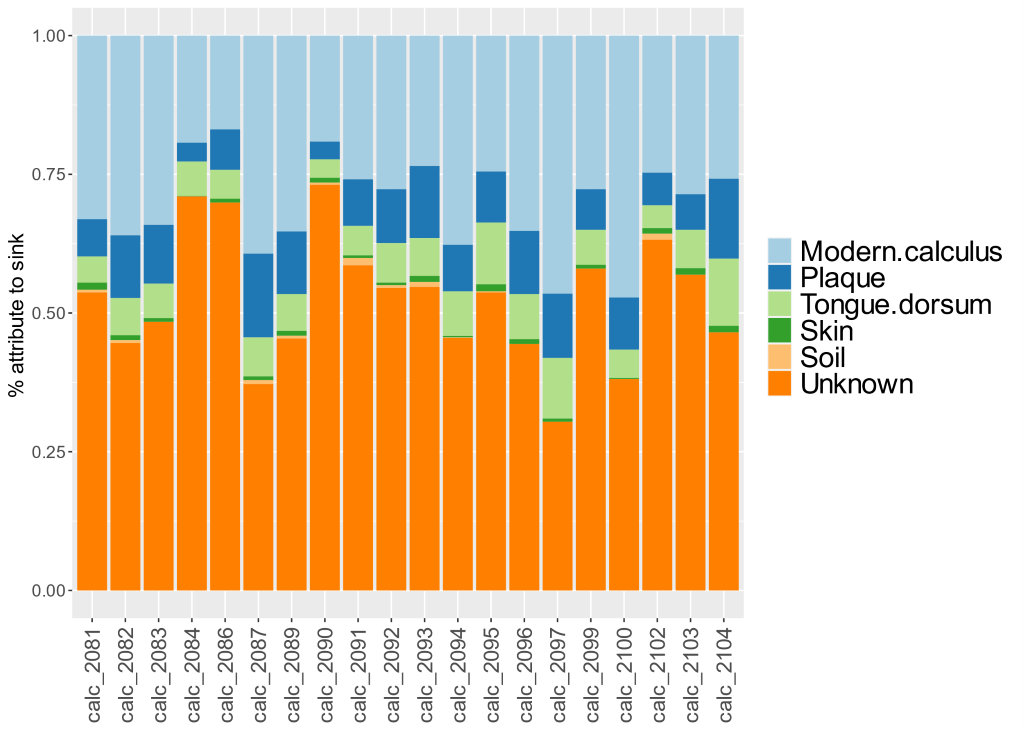


**Figure S3**. Sourcetracker[9] analysis for all Trentino-South Tyrolean calculus samples indicating the proportions of each source dataset based on species-level taxonomic abundances.


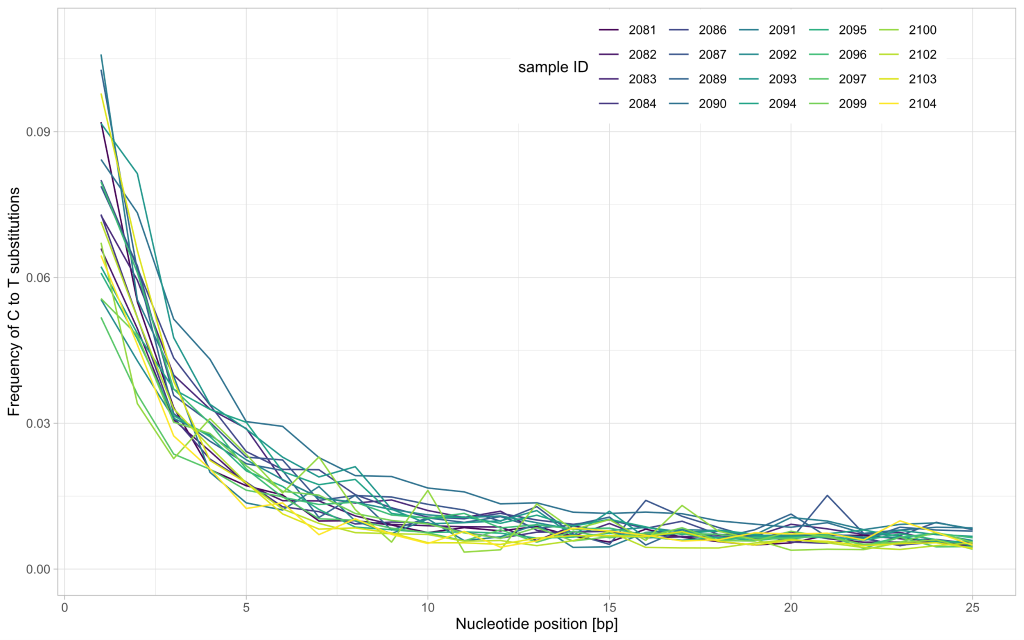


**Figure S4**. Frequency of C to T transitions in reads mapped to the highest abundant taxa present in the Trentino-South Tyrolean calculus dataset, *Desulfobulbus* oral taxon 041 (GCA_000349345.1), estimated by MapDamage[10].

**
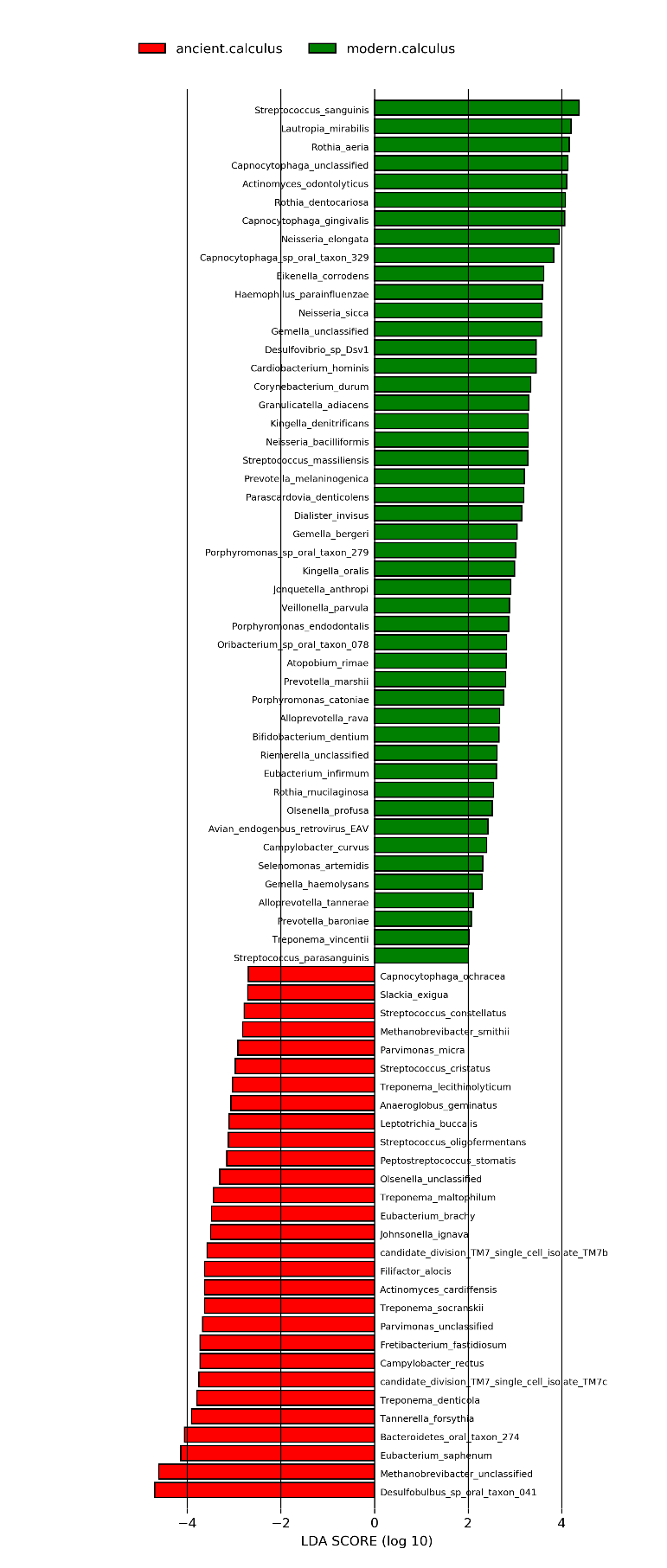
**

**Figure S5**. Species level linear discriminant analysis (LDA) effect size (LEfSe)[11] between ancient and modern calculus.


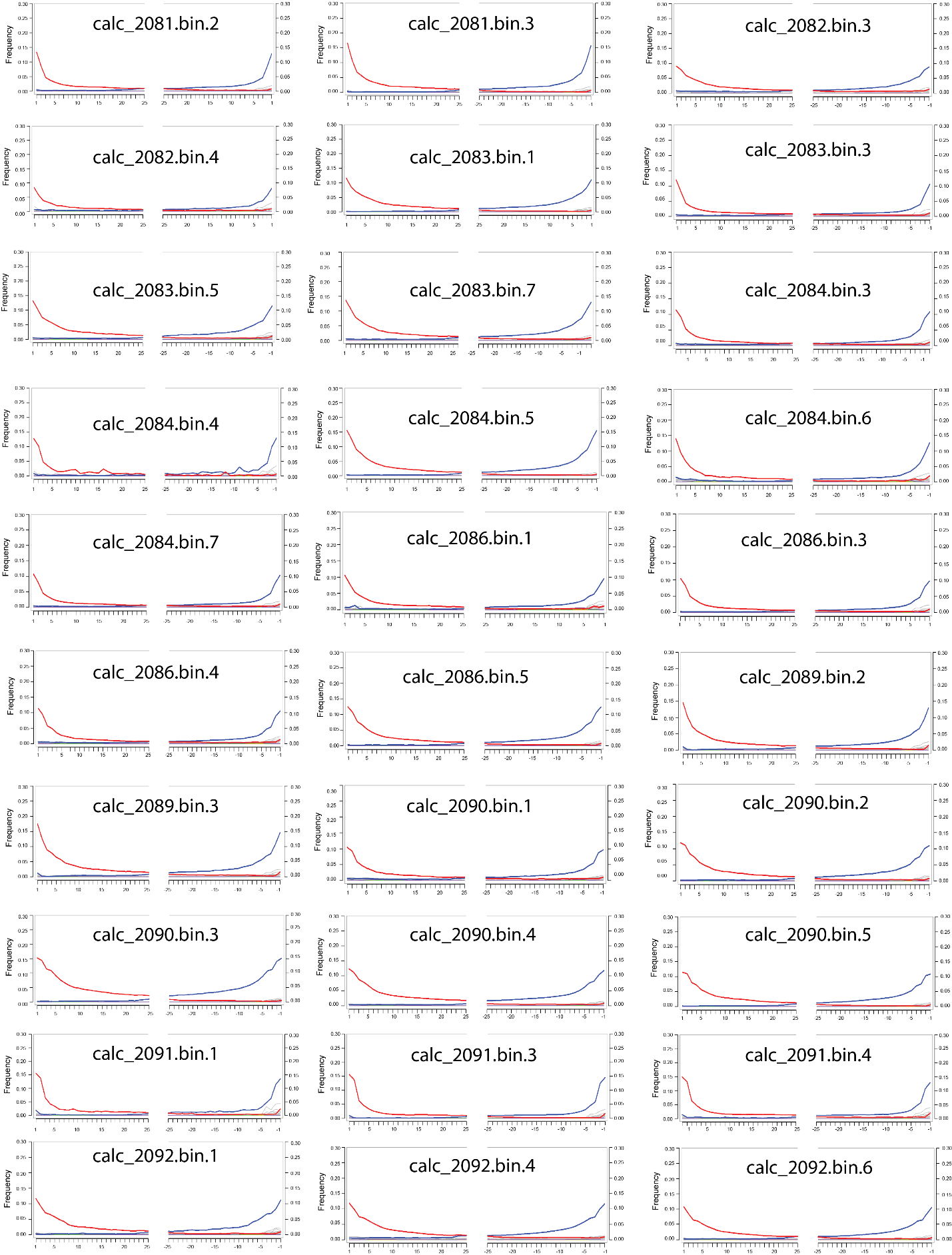


**
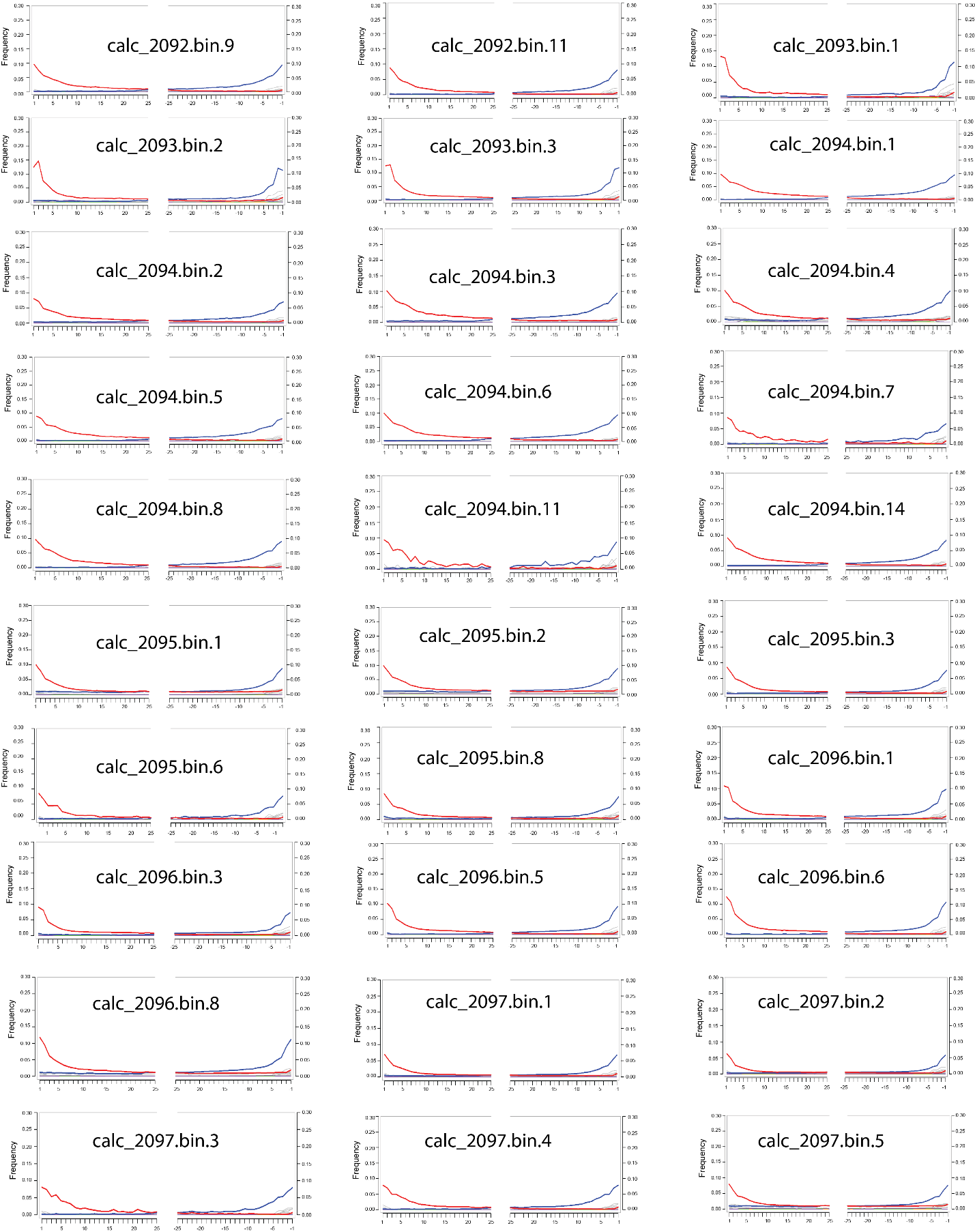

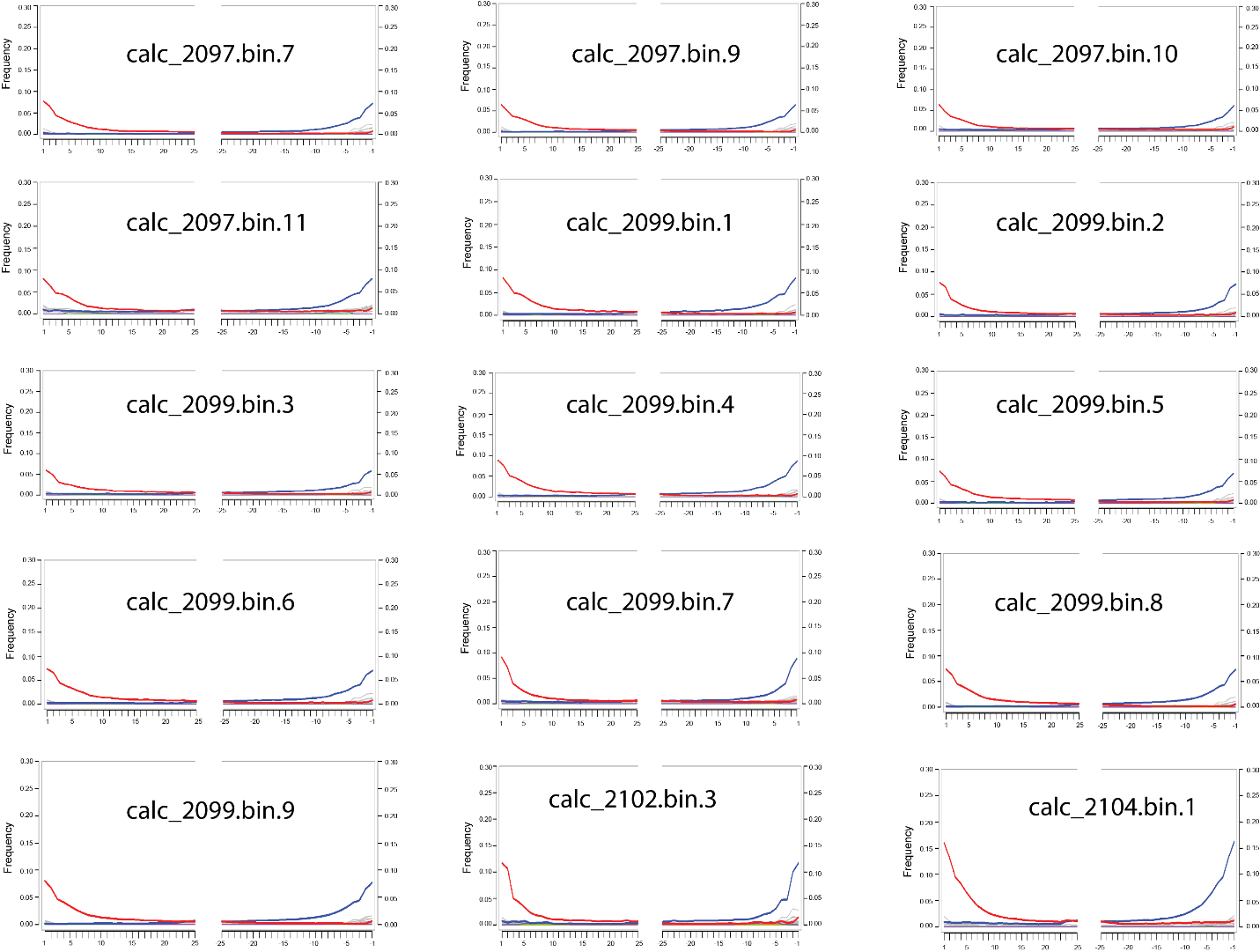
Figure S6**. Authentication of endogenous origin for 76 newly reconstructed microbial genomes.

**
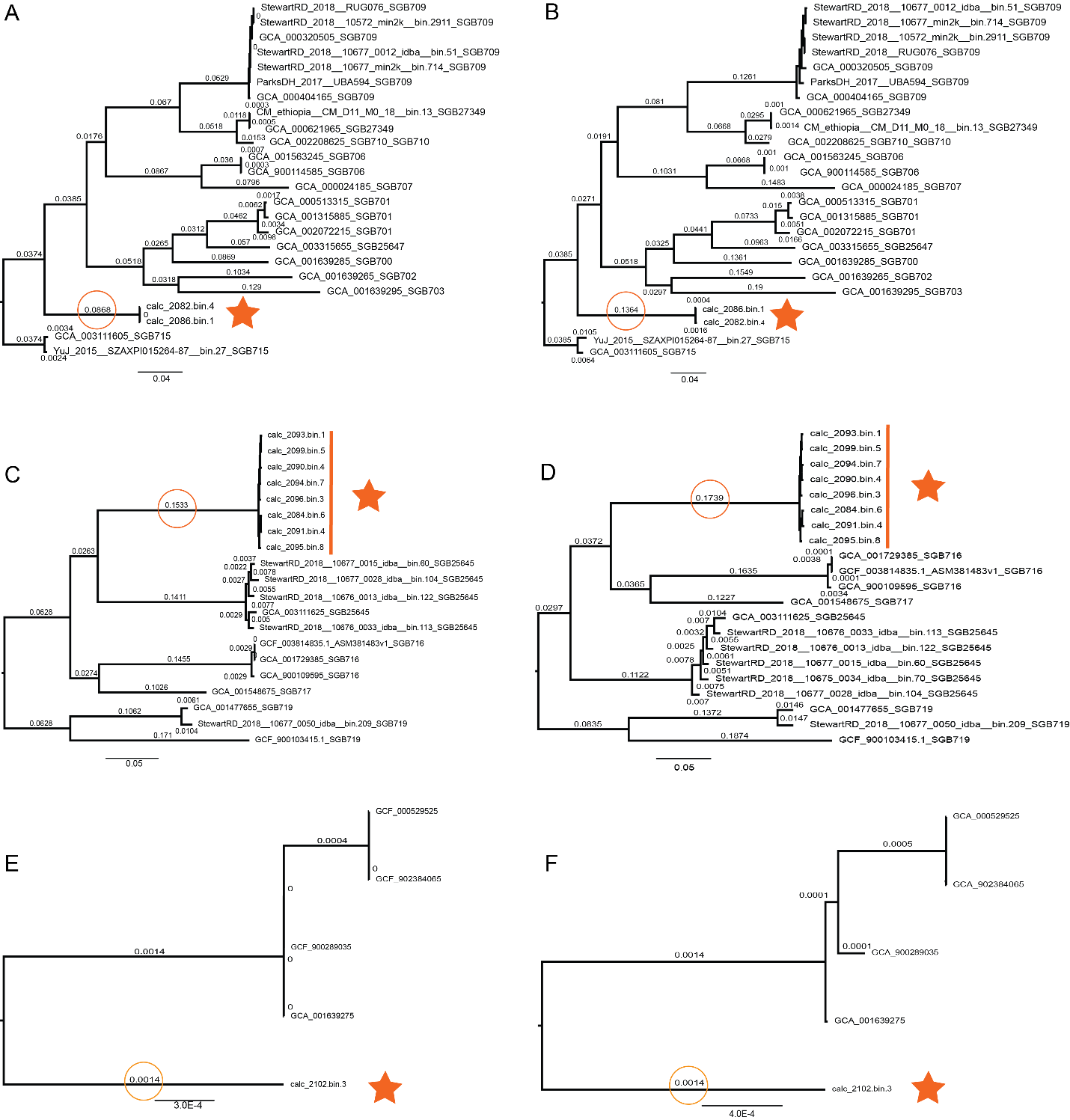
Figure S7**. Resolution-improved phylogeny of subtrees based on the core and whole genome. (A), (C), and (E) indicate subtree1, subtree2, and subtree3, based on core gene alignment generated by MAFFT [12] , respectively. (B), (D), and (F) indicate subtree1, subtree2, and subtree3, based on the whole genome alignment, respectively (see Methods). The orange star highlights ancient genomes and the red circle emphasizes the long branch length.

**
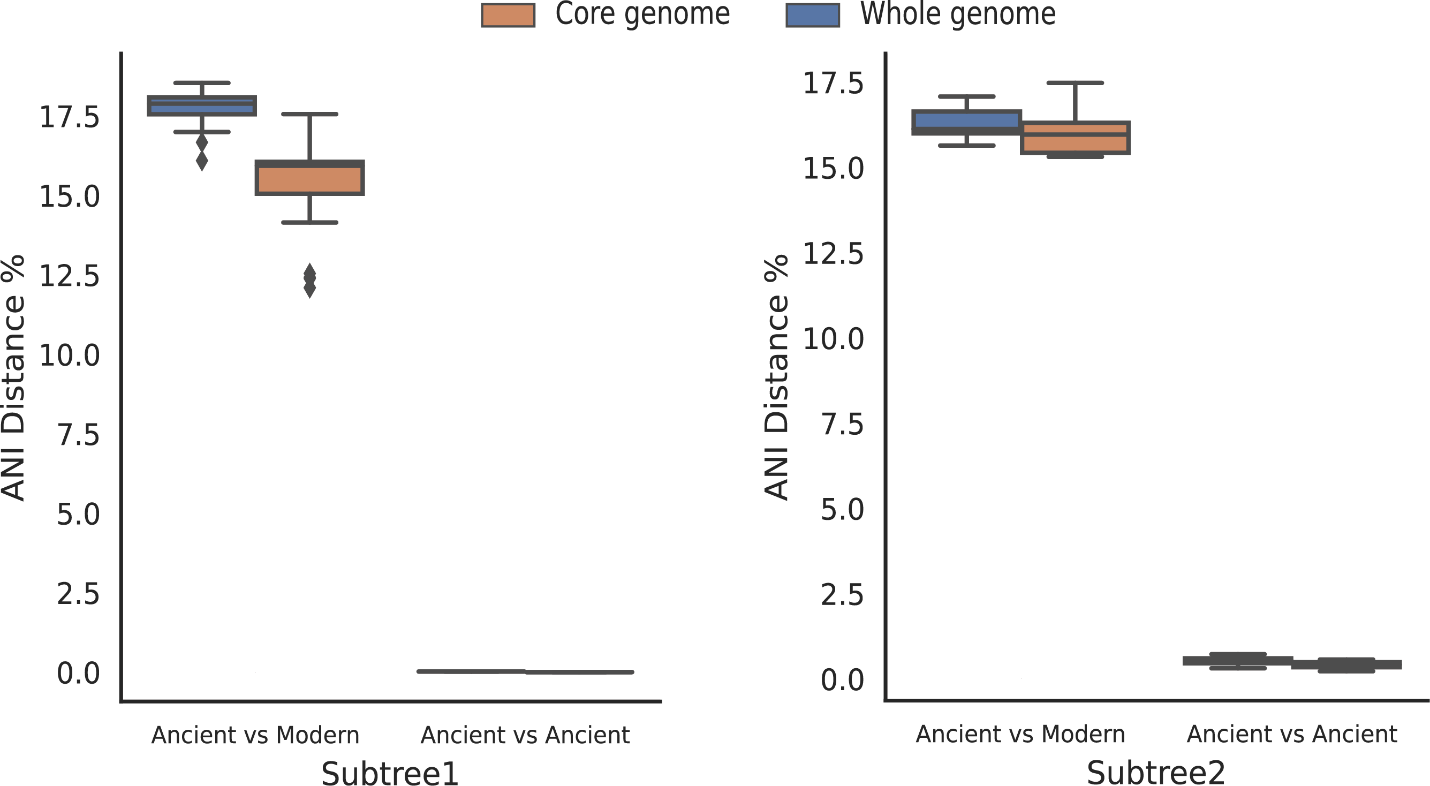
**

**Figure S8.** Genetic distance pairwise distances within ancient genomes and between ancient and modern genomes in subtree 1 (TS-1) and subtree 2 (TS-2) respectively, measured by ANI.


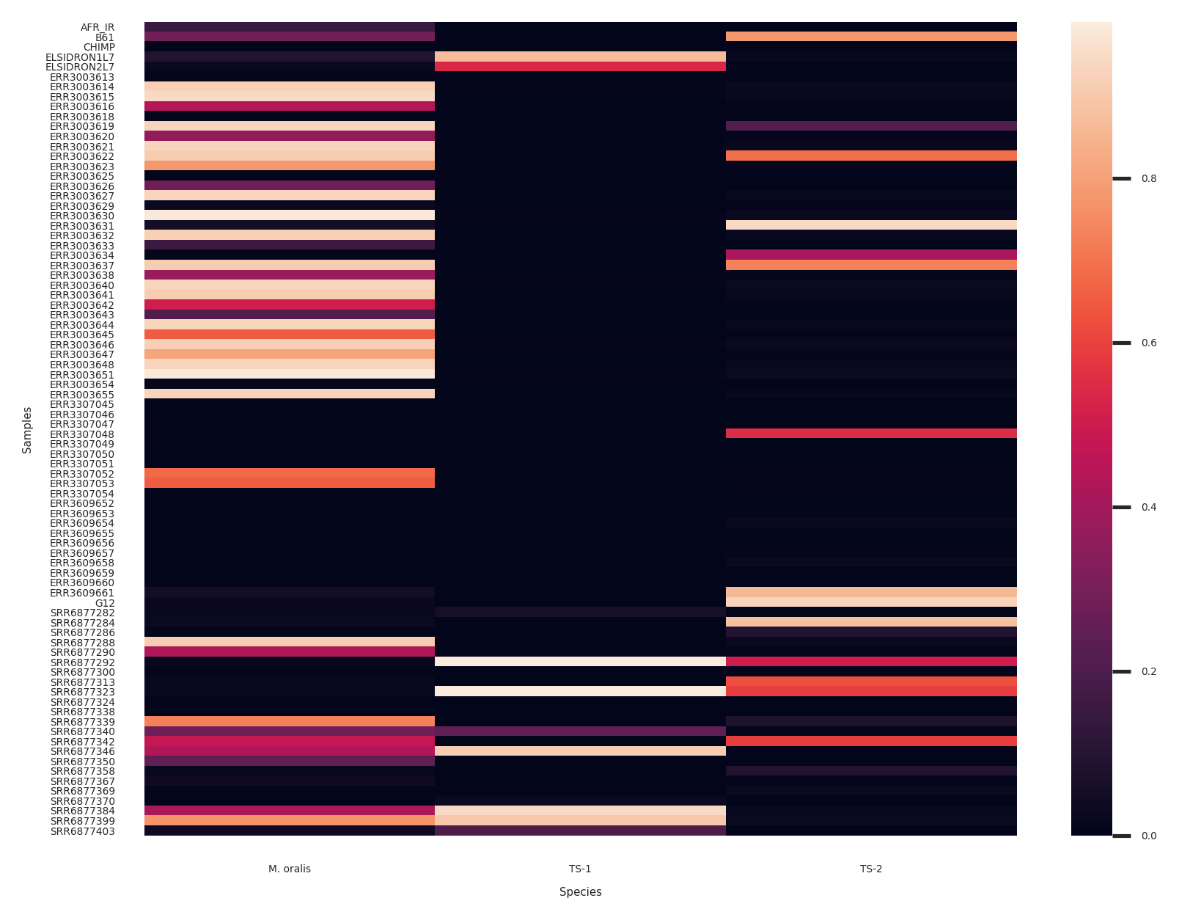


**Figure 9.** Alignment-based assessment for species coverage across publicly available ancient and modern calculus metagenomic samples. The heatmap shows the percentage of the genome covered at >3X depth. Reference genome GCA_001639275 represents *M. oralis*, newly reconstructed genome calc_2084.bin.1 represents *TS-1*, and calc_2094.bin.7 was used for another species *TS-2*.

**
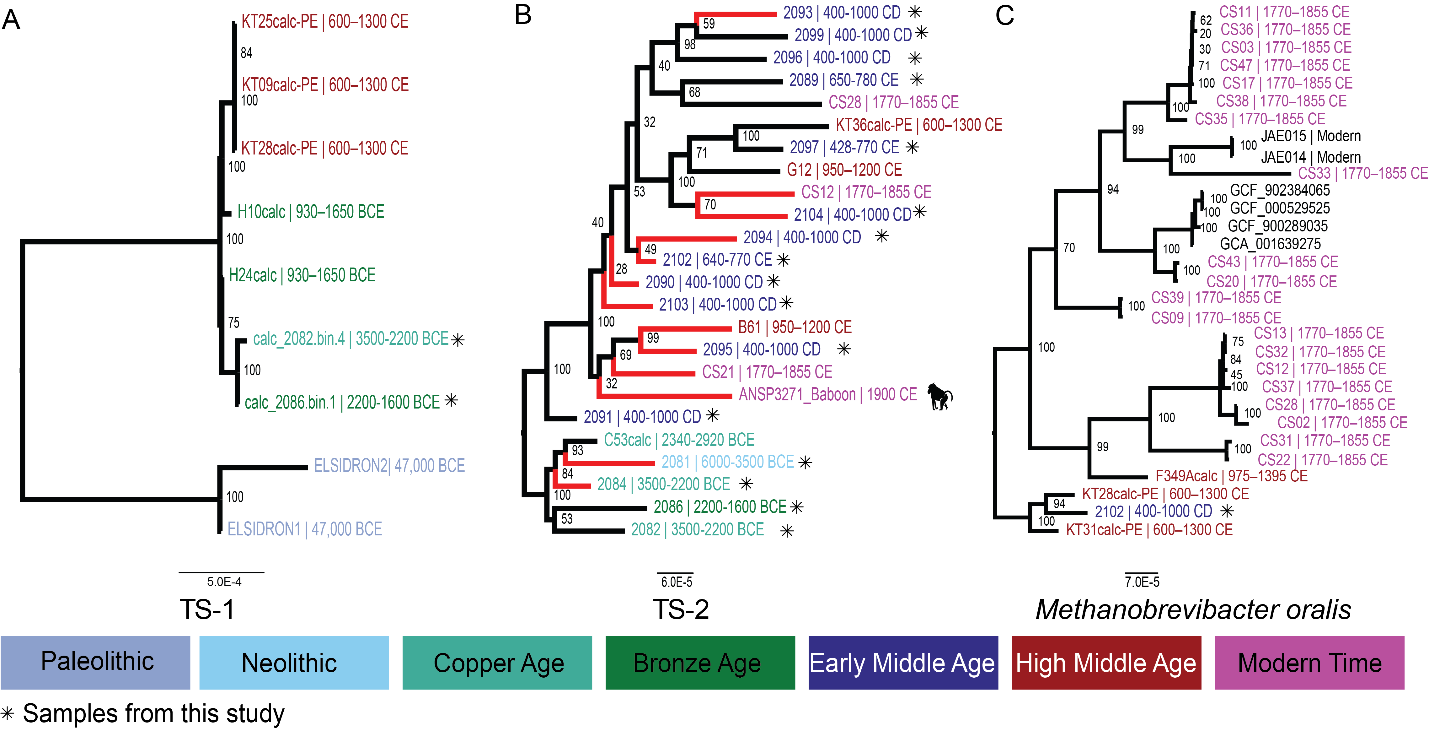
Figure 10**. Strain-resolved phylogeny of newly discovered *Methanobrevibacter* species TS-1 (A), TS-2 (B) and *Methanobrevibacter* *oralis* (C), reconstructed using draft ancient genomes from alignment-based approach. Branches variable in phylogenetic position from those in Figure 4D are marked as red.


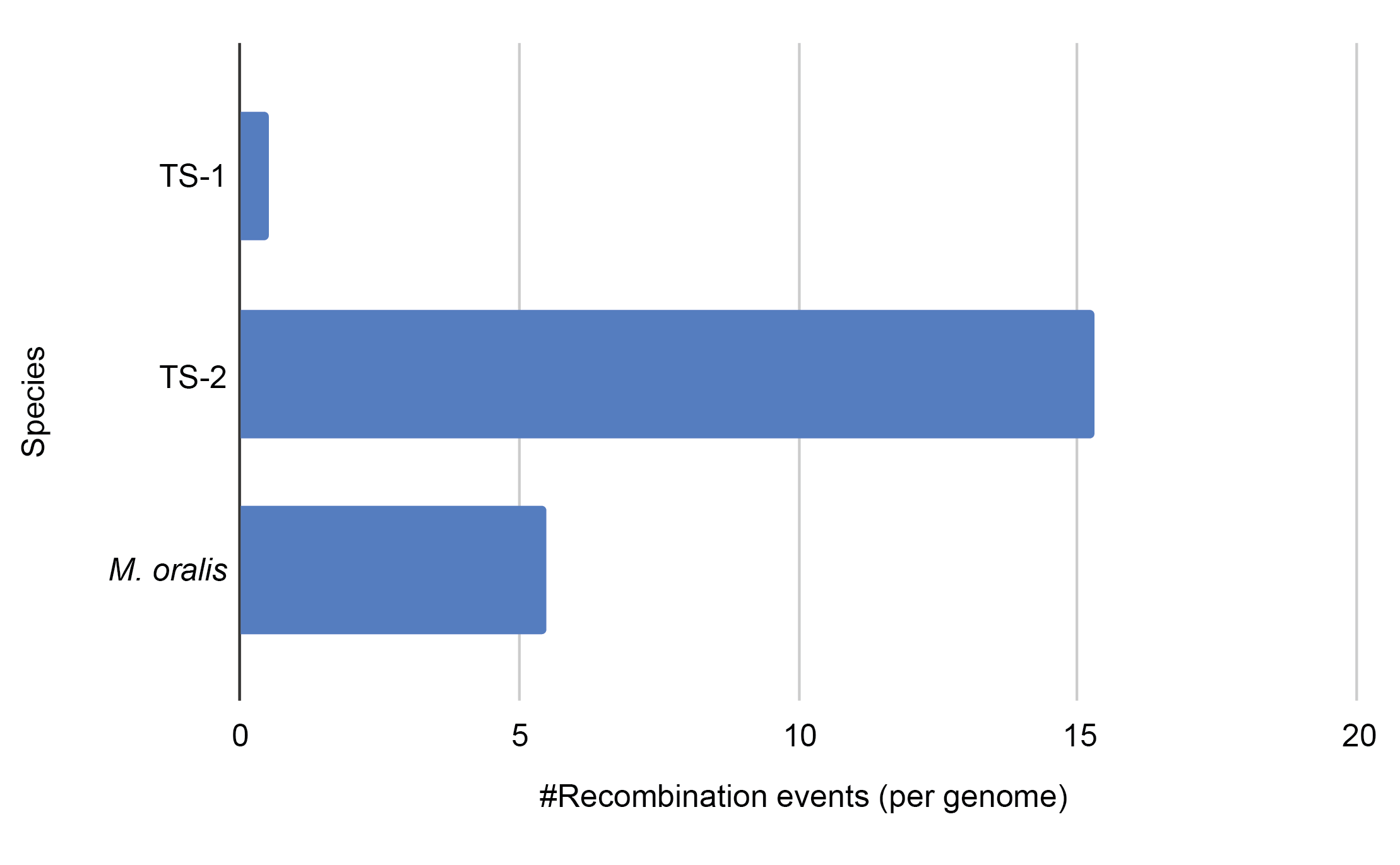


**Figure S11**. The number of recombination events (normalized by the total number of genomes in the alignment) detected in TS-1, TS-2 and *M. oralis*, using ClonalFrameML [13].

**
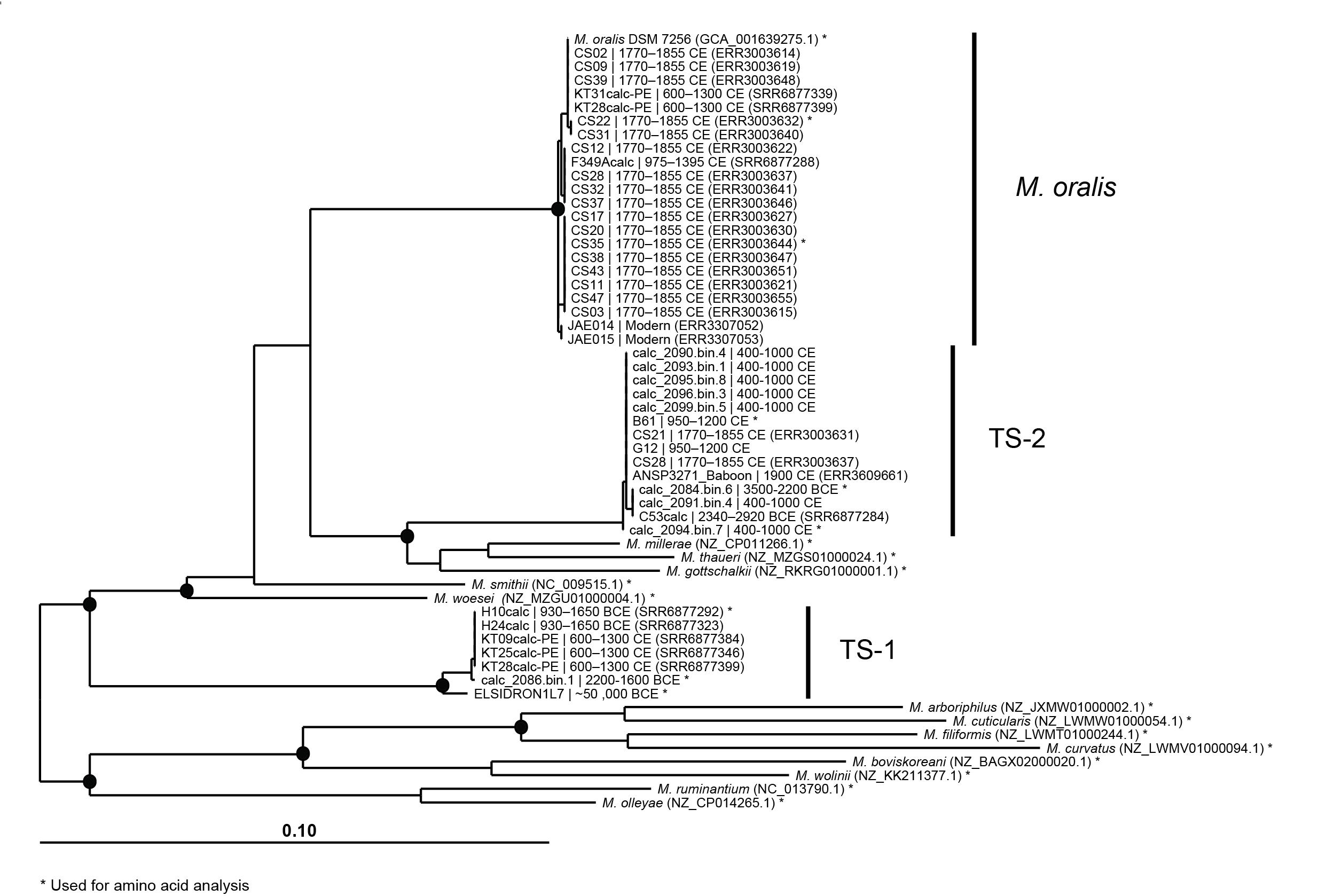
**

**Figure S12**. Maximum likelihood tree based on a *mcrA* nucleic acid sequence alignment. Sequences marked with an asterisk indicate *mcrA* sequences used for amino acid analysis. Black circles symbolize parsimony and neighbour joining bootstrap support (>60%) based on 100 and 1000 iterations, respectively. The scale bar depicts 0.1 substitutions per residue.

Methyl-coenzyme M reductase I subunit alpha

Name: Mor Len: GCA_001639275.1, *M. oralis* DSM 7256

Name: CS2 Len: CS22 ERR3003632 Moralis2

Name: CS3 Len: CS35 ERR3003644 Moralis2

Name: c86 Len: SGB39694_calc_2086

Name: ELS Len: ELSIDRON1L7 SGB39694

Name: H10 Len: H10calc SRR6877292 SGB39694

Name: c84 Len: SGB39695_calc_2084

Name: c94 Len: SGB39695_calc_2094

Name: B61 Len: B61 SGB39695

Name: Mw1 Len: NZ_MZGU01000004.1 M. woesei strain DSM 11979

Name: Msm Len: NC_009515.1 M. smithii ATCC 35061

Name: Mmi Len: NZ_CP011266.1 M. millerae strain SM9

Name: Mth Len: NZ_MZGS01000024.1 M. thaueri strain DSM 11995

Name: Mgo Len: NZ_RKRG01000001.1 M. gottschalkii DSM 11977

Name: Mol Len: NZ_CP014265.1 M. olleyae strain YLM1

Name: Mru Len: NC_013790.1 M. ruminantium M1

Name: Mw2 Len: NZ_KK211377.1 M. wolinii SH

Name: Mbo Len: NZ_BAGX02000020.1 M. boviskoreani JH1

Name: Mar Len: NZ_JXMW01000002.1 M. arboriphilus JCM 13429

Name: Mfi Len: NZ_LWMT01000244.1 M. filiformis strain DSM 11501

Name: Mc1 Len: NZ_LWMV01000094.1 M. curvatus strain DSM 11111

Name: Mc2 Len: NZ_LWMW01000054.1 M. cuticularis strain DSM 11139

1 11 21 31 41 51 61 71 81 91 100

| | | | | | | | | | |

Mor MADKKFLDAM KQKFSEDPTE KRTQFYNMGG WKQSERKTAF VNEGKKIAEE RGIPMYNPDI GTPLGQRALM SYQLSTTDTF VEGDDLHFIN NSAIQQAWDD

CS2 MADKKFLDAM KQKFSEDPTE KRTQFYNMGG WKQSERKTAF VNEGKKIAEE RGIPMYNPDI GTPLGQRALM SYQLSTTDTF VEGDDLHFIN NSAIQQAWDD

CS3 MADKKFLDAM KQKFSEDPTE KRTQFYNMGG WKQSERKTAF VNEGKKIAEE RGIPMYNPDI GTPLGQRALM SYQLSTTDTF VEGDDLHFIN NSAIQQAWDD

SGB MADKRFLEAM KKKFKEDPTD KRTSFYNMDG WKQSERKCAF VKEGEEIAKE RGIPMYNPDI GNPLGQRALM SYQLSTTDTF VEGDDLHFIN NAAIQQAWDD

ELS MADKRFLEAM KKKFKEDPTD KRTSFYNMDG WKQSERKCAF VKEGEEIAKE RGIPMYNPDI GNPLGQRALM SYQLSTTDTF VEGDDLHFIN NAAIQQAWDD

H10 MADKRFLEAM KKKFKEDPTD KRTSFYNMDG WKQSERKCAF VKEGEEIAKE RGIPMYNPDI GNPLGQRALM SYQLSTTDTF VEGDDLHFIN NAAIQQAWDD

c84 MADKKFLDAM KQKFSEDPTE KTTQFYNMGG WTQSERKTAF VNEGKEIAEK RGIPMYNPDI GTPLGQRALM SYQLSTTDTF VEGDDLHFIN NAAIQQAWDD

c94 MADKKFLDAM KQKFSEDPTE KTTQFYNMGG WTQSERKTAF VNEGKEIAEK RGIPMYNPDI GTPLGQRALM SYQLSTTDTF VEGDDLHFIN NAAIQQAWDD

B61 MADKKFLDAM KQKFSEDPTE KTTQFYNMGG WTQSERKTAF VNEGKEIAEK RGIPMYNPDI GTPLGQRALM SYQLSTTDTF VEGDDLHFIN NAAIQQAWDD

Mw1 MADKKFLDAM KQKFSEDPTD DRTTFYNMGG WKQSERKSAF VKEGQEIAEK RGIPMYNPDI GTPLGQRALM SYQLSTTDTF VEGDDLHFIN NSAIQQAWDD

Msm MADKKFLDAM KNKFSEGPTD KRTTFYNMGG WKQSERKSAF VKEGKEIAEK RGIPMYNPDI GTPLGQRALM SYQLSTTDTY VEGDDLHFIN NAAIQQAWDD

Mmi MADKKFLDAM KQKFSEDPTE KTTQFYNMGG WTQSERKTAF VNEGKEIAEK RGIPMYNPDI GTPLGQRALM SYQLSTTDTF VEGDDLHFIN NAAIQQAWDD

Mth MADKKFLDAM KKKFTEDPTE KRTQFYNMGG WTQSERKTAF VNEGKEIAAK RGIPMYNPDI GSPLGQRALM SYQLSTTDTF VEGDDLHYIN NAAIQQAWDD

Mgo MADKKFLNAM KQKFSEDPTE KSTQFYNMGG WTQSERKTAF VNEGKEIAEK RGIPMYNPDI GTPLGQRALM SYQLSTTDTF VEGDDLHFIN NAAIQQAWDD

Mol MADKKFLDAM TKKFKEAPDE RTTTFYNMGG WTQSERKTEF VNEGKSIAEK RGIPMYNPDI GNPLGQRALM SYQLSGTDTF VEGDDLHFIN NAAMQQAWDD

Mru MADKKFLDAM TKKFKEAPEE KTTTFYNMGG WTQSERKTEF VNEGKAIAEA RGIPMYNPDI GNPLGQRALM SYQLSGTDTF VEGDDLHFIN NAAMQQAWDD

Mw2 MADKKFIDAL NKKFKESPED KTTTFYNLGG WRQSERKTEF YEAGKEIAEK RGIPQFNPDV GSPLGQRTLM PYQLSGTDTF VEGDDLHFIN NAAIQQMWDD

Mbo MADKKFYEAL NKKFKESPED KTTTFYNLGG WKQSERKTQF YEAGKEIAEE RGIPMYNPDI GSPLGQRTLM PYQLSTTDTF VEGDDLHFIN NAAIQQLWDD

Mar MADKKFIEAL NKKFKEDPEE KTTTFYNLGG WKQSERKTEF ANAGKEIAEK RGIPQYNPDV GSPLGQRALM PYQVSTTDTY VEGDDFHFVN NAAIQQMWDD

Mfi MADKKFIQAL DKKFKEAPED KTTTFYNLGG WKQSERKTEF YNAGKEIAEK RGIPQYNPDV GSPLGQRTLM PYQVSTTDTY VEGDDFHFVN NAAIQQMWDD

Mc1 MADKKFIDAL NKKFKEDPTE KSTTFYNLGG WTQSERKTEF ANAGKEIAEK RGIPQYNPDV GSPLGQRALM PYQVSTTDTY VEGDDFHFVN NAAIQQMWDD

Mc2 MANKKFIEAL NKKFKESPEE KTTTFYNLGG WKQSERKSEF AKEGKEIATK RGIPQYNPDV GSPLGQRALM PYQVSTTETF VEGDDFHFVN NAAIQQMWDD

101 111 121 131 141 151 161 171 181 191 200

| | | | | | | | | | |

Mor IRKTVIVGLN TAHNVLEKRL GIEVTPETIT HYLETVNHAM PGAAVVQEHM VETDPLVVSD SYVKVFTGDD ELADEIDSAF VLDINKEFPE EQAEALKAEV

CS2 IRKTVIVGLN TAHNVLEKRL GIEVTPETIT HYLETVNHAM PGAAVVQEHM VETDPLVVSD SYVKVFTGDD ELADEIDSAF VLDINKEFPE EQAEALKAEV

CS3 IRKTVIVGLN TAHNVLEKRL GIEVTPETIT HYLETVNHAM PGAAVVQEHM VETDPLVVSD SYVKVFTGDD ELADEIDSAF VLDINKEFPE EQAEALKAEV

C86 IRRTVIVGLN TAHNVLEKRL GIEVTPETIT EYLEIVNHAM PGAAVVQEHM VETDPLVVSD SYVKVFTGDD ELADEIDSAF VLDINKEFND EQANALKNEV

ELS IRRTVIVGLN TAHNVLEKRL GIEVTPETIT EYLEIVNHAM PGAAVVQEHM VETDPLVVSD SYVKVFTGDD ELADEIDSAF VLDINKEFND EQANALKNEV

H10 IRRTVIVGLN TAHNVLEKRL GIEVTPETIT EYLEIVNHAM PGAAVVQEHM VETDPLVVSD SYVKVFTGDD ELADEIDSAF VLDINKEFND EQANALKNEV

c84 IRRTVIVGLN TAHNVLEKRL GIEVTPETIT NYLEIVNHAM PGAAVVQEHM VETDPLVVQD SYVKVFTGDD ELADEIDSAF VLDINKEFPE EQAEALKAEV

c94 IRRTVIVGLN TAHNVLEKRL GIEVTPETIT NYLEIVNHAM PGAAVVQEHM VETDPLVVQD SYVKVFTGDD ELADEIDSAF VLDINKEFPE EQAEALKAEV

B61 IRRTVIVGLN TAHNVLEKRL GIEVTPETIT NYLEIVNHAM PGAAVVQEHM VETDPLVVQD SYVKVFTGDD ELADEIDSAF VLDINKEFPE EQAEALKAEV

Mw1 IRKTVIVGLN TAHNVLEKRL GIEVTPETIT EYLETVNHAM PGAAVVQEHM VETDPLVVSD SYVKVFTGDD ELADEIDSAF VLDINKEFPE EQAEALKAEV

Msm IRRTVIVGLN TAHNVLEKRL GIEVTPETIT EYLETVNHAM PGAAVVQEHM VETDPLVVQD SYVKVFTGDD ELADEIDSAF VLDINKEFNE EQAAALKEEV

Mmi IRRTVIVGLN TAHNVLEKRL GIEVTPETIT NYLETVNHAM PGAAVVQEHM VETDPLVVQD SYVKVFTGDD ELADEIDSAF VLDINKEFPE EQAEALKAEV

Mth IRRTVIVGLN TAHNVLEKRL GIEVTPETIT NYLETVNHAM PGAAVVQEHM VETDPLVVQD SYVKVFTGDD ELADEIDSAF VLDINKEFPE EQAEALKAEV

Mgo IRRTVIVGLN TAHNVLEKRL GIEVTPETIT NYLETVNHAM PGAAVVQEHM VETDPLLVAD SYVKVFTGDD ELADEIDSAF VLDINKEFNE EQAEALKAEV

Mol IRRTVIVGLN TAHNVLEKRL GMEVTPETIT NYLEVVNHAM PGAAVIQEHM VETNPLLVED SYVKIFTGDD DLADEIDAAF VLDINKEFPE EQAEVLKSEV

Mru IRKTVIVGLN TAHNVLEKRL GMEVTPETIT NYLEVVNHAM PGAAAVQEHM VETNPLLVDD SYVKVFTGDD DLAAEIDPAF VLDINKEFPE EQAEALKAEV

Mw2 IRRTVIVGLN TAHNVLERRL GMEVTPETIT RYLETLNHAM PGGAVVQEHM VEINPYLAAD SYVKVFTGDD ELADEIDKCY VLDINKEFPE EQAEALKAEV

Mbo IRRTVLVGLN TAHNVLQRRL GMEVTPETIT NYLETVNHAM PGAAVVQEHM VETNPYLVSD SYVKIFTGDD ELADEIDQCY VLDINKEFPD DQAAQLKAEV

Mar IRRTVIVGLN TAHNVLEKRL GMEVTPETIT EYLETVNHAM PGAAVVQEHM VETNPSLVAD SYVKVFTGDD ELADEIDSAF VLDINKEFPE DQAEALKAEV

Mfi IRRTVVVGLN TAHTVLEKRL GIEVTPETIT NYLETVNHAM PGAAVVQEHM VETNPLLVAD SYVKVFTGDD ELADEIDSAF VLDINKEFPE DQAAVLKEEV

Mc1 IRRTVIVGLN TAHNVIEKRL GMEVTPETIT HYLETVNHAM PGAAVVQEHM VETNPLLVAD SYVKIFTGDD ELADEIDKAY VLDINKEFNE EQAAALKAEV

Mc2 IRRTVIVGLN TAHTVLEKRL GIEVTPETIT EYLETVNHAM PGAAVVQEHM VETNPALVAD SYVKIFTGDD EVADEIDSAF VLDINKEFSK EQAEALKAEV

201 211 221 231 241 251 261 271 281 291 300

| | | | | | | | | | |

Mor GGSVWQAVRI PAIVGRVCDG GTTSRWSAMQ IGMSMISAYN QCAGEGATGD FAYASKHAEV IHMGTYLPVR RARAENELGG VPFGFMADIC QGTRVHDDPV

CS2 GGSVWQAVRI PAIVGRVCDG GTTSRWSAMQ IGMSMISAYN QCAGEGATGD FAYASKHAEV IHMGTYLPVR RARAENELGG VPFGFMADIC QGTRVHDDPV

CS3 GGSVWQAVRI PAIVGRVCDG GTTSRWSAMQ IGMSMISAYN QCAGEGATGD FAYASKHAEV IHMGTYLPVR RARAENELGG VPFGFMADIC QGTRVHDDPV

C86 GGSVWQVVRI PSVVGRICDG GTTSRWSAMQ IGMSMISAYN QCAGEGATGD FAYASKHAEV VHMGTYLPVR RARAENELGG VPFGFMADIC QASRVNDDPV

ELS GGSVWQVVRI PSVVGRICDG GTTSRWSAMQ IGMSMISAYN QCAGEGATGD FAYASKHAEV VHMGTYLPVR RARAENELGG VPFGFMADIC QASRVNDDPV

H10 GGSVWQVVRI PSVVGRICDG GTTSRWSAMQ IGMSMISAYN QCAGEGATGD FAYASKHAEV VHMGTYLPVR RARAENELGG VPFGFMADIC QASRVNDDPV

c84 GGAVWQAVRI PSIVGRVCDG GTTSRWSAMQ IGMSMISAYN QCAGEGATGD FAYASKHAEV VHMGTYLPVR RARAENELGG VPFGFMADIC QASRVNDDPV

c94 GGAVWQAVRI PSIVGRVCDG GTTSRWSAMQ IGMSMISAYN QCAGEGATGD FAYASKHAEV VHMGTYLPVR RARAENELGG VPFGFMADIC QGSRVYDDPV

B61 GGAVWQAVRI PSIVGRVCDG GTTSRWSAMQ IGMSMISAYN QCAGEGATGD FAYASKHAEV VHMGTYLPVR RARAENELGG VPFGFMADIC QGSRVYDDPV

Mw1 GGSIWQAVRI PGIVGRVCDG GTTSRWSAMQ IGMSMISAYN QCAGEGATGD FAYASKHAEV IHMGTYLPIR RARAENELGG VPFGFMADIC QATRLYDDPV

Msm GGSVWQAVRI PAIVGRVCDG GTTSRWSAMQ IGMSMISAYN QCAGEGATGD FAYASKHAEV IHMGTYLPVR RARAENELGG VPFGFMADIC QSSRVNDDPV

Mmi GGAVWQAVRI PSIVGRVCDG GTTSRWSAMQ IGMSMISAYN QCAGEGATGD FAYASKHAEV VHMGTYLPVR RARAENELGG VPFGFMADIC QASRVSDDPV

Mth GGAIWQAVRI PSIVGRVCDG GNTSRWSAMQ IGMSMISAYN QCAGEGATGD FAYASKHAEV IHMGTYLPVR RARAENELGG VPFGFMADIC QGSRAYDDPV

Mgo GSAVWQAVRI PSIVGRVCDG GTTSRWSAMQ IGMSMISAYN QCAGEGATGD FAYASKHAEV VQMGTYLPVR RARAANELGG VPFGFMADIC QGSRVYDDPV

Mol GGAIWQIVRI PSVVGRVCDG GTTSRWSAMQ IGMSMISAYG QCAGEGATGD FAYASKHAEV IGMGTYLPIR RARADNELGG VPFGFMADIC QATRVSDDPV

Mru GGAIWQIVRV PSVVGRVCDG GTTSRWSAMQ IGMSMISAYG QCAGEGATGD FAYASKHAEV IGMGTYLPIR RARAGNELGG VPFGFMADIC QATRVTDDPV

Mw2 GDKIWQAVRI PSVVGRVCDG GVTSRWSAMQ IGMSMISAYN QCAGEGATGD FAYASKHAEV IQMGTPLPQR RARAANEPGG IPFGYMADIV QSSRVNDDPV

Mbo GDKIWQVVRV PSLVGRVCDG GTTSRWSAMQ IGMSMISAYN QCAGEGATGD FAFATKHAEV IQMGAPLPQR RARAANEPGG VPFGYIADIV QSSRVNDDPV

Mar GDKVWQAVRI PTIVGRVCDG GTTSRWSAMQ IGMSMISAYN QCAGEGATGD FAYASKHAEV IHMGTYLPVR RARAENEPGG IAFGFLADMV QSTRVNEDPV

Mfi GDKIWQVVRI PSIVGRVCDG GTTSRWSAMQ IGMSMISSYN QCAGEGATGD FAYASKHAEV IHMGTYLPVR RARAENEPGG IPFGVMADIN QSSRVNDDPV

Mc1 GDNIWQVVRI PSIVGRVCDG GTTSRWSAMQ IGMSMISAYN QCAGEGATGD FAYASKHAEV IHMGTYLPVR RARAENEPGG VAFGYMADIC QSSRINDDPV

Mc2 GDKIWQVVRI PSIVGRVCDG GTTSRWSAMQ IGMSMISAYN QCAGEGATGD FAYASKHAEV IHMGTYLPVR RARAENEPGG IPFGYMADIC QSSRVNDDPV

301 311 321 331 341 351 361 371 381 391 400

| | | | | | | | | | |

Mor RSTLEVVALG AALYDQIWLG SYMSGGVGFT QYATAAYTDN VLDDFTYMTV LDVGTEVAFY ALEQYEEYPL ETGGSQRSSV ISAAAGASTA FATAWYLSMY

CS2 RSTLEVVALG AALYDQIWLG SYMSGGVGFT QYATAAYTDN VLDDFTYMTV LDVGTEVAFY ALEQYEEYPL ETGGSQRSSV ISAAAGASTA FATAWYLSMY

CS3 RSTLEVVALG AALYDQIWLG SYMSGGVGFT QYATAAYTDN VLDDFTYMTV LDVGTEVAFY ALEQYEEYPL ETGGSQRSSV ISAAAGASTA FATAWYLSMY

C86 RSSLEVVGLA SALYDQIWLG SYMSGGVGFT QYATAAYTDD VLDDFTYMTV LDVGTEVTFY SLEQYEEYPL ETGGSQRASV VSAAAGCSTA FATAWYLAMY

ELS RSSLEVVGLA SALYDQIWLG SYMSGGVGFT QYATAAYTDD VLDDFTYMTV LDVGTEVTFY SLEQYEEYPL ETGGSQRASV VSAAAGCSTA FATAWYLAMY

H10 RSSLEVVGLA SALYDQIWLG SYMSGGVGFT QYATAAYTDD VLDDFTYMTV LDVGTEVTFY SLEQYEEYPL ETGGSQRASV VSAAAGCSTA FATAWYLAMY

c84 RTSLEVVALG AALYDQIWLG SYMSGGVGFT QYATAAYTDN VLDDFTYMTV LDVGTEVAFY ALEQYEEYPL ETGGSQRSSV ISAAAGCSTA FATAWYLSMY

c94 RTSLEVVALG AALYDQIWLG SYMSGGVGFT QYATAAYTDN VLDDFTYMTV LDVGTEVAFY ALEQYEEYPL ETGGSQRSSV ISAAAGCSTA FATAWYLSMY

B61 RTSLEVVALG AALYDQIWLG SYMSGGVGFT QYATAAYTDN VLDDFTYMTV LDVGTEVAFY ALEQYEEYPL ETGGSQRSSV ISAAAGCSTA FATAWYLSMY

Mw1 RSSLEVVALG AALYDQIWLG SYMSGGVGFT QYATAAYTDN VLDDFSYMTV LDVGSEVTFY SLEQYEEYPL ETGGSQRAAV VSAAAGISTA FATAWYLAQY

Msm RSTLDVVALG AALYDQIWLG SYMSGGVGFT QYATAAYTDD VLDDFTYMTV LDVGSEVSFY ALEQYEEYPL ETGGSQRASV ISAAAGCSTA FATAWYLGMY

Mmi KTSLEVVALG AALYDQIWLG SYMSGGVGFT QYATAAYTDN VLDDFTYMTV LDVGTEVAFY ALDQYEEYPL ETGGSQRASV ISAAAGCSTA FATAWYLAMY

Mth RSTLEVVALG AALYDQIWLG SYMSGGVGFT QYATAAYTDN VLDDFTYMTV LDVGTEVAFY ALEQYEEYPL ETGGSQRASV ISAAAGCSTA FATAWYLSMY

Mgo RQTLEVVALG AALYDQIWLG SYMSGGVGFT QYATAAYTDN VLDDFTYMTV LDVGSEVAFY ALDQYEEYPL ETGGSQRASV ISAAAGCSTA FATGWYLSMY

Mol KSSLEVVALG AALYDQIWLG SYMSGGVGFT QYATAAYTDN VLDDFSYMTV LDVGSEVAFY SLEQYEEYPL ETGGSQRAAV ISAASGISTA FATAWYLAQY

Mru ESALEVVALG AALYDQIWLG SYMSGGVGFT QYATAAYTDN VLDDFSYMTV LDVGSAVTFY SLEQYEEYPL ETGGSQRAAV VSAASGISTA FATAWYLAQY

Mw2 KISLDVVAAG AALYDQVWLG SYMSGGVGFT QYATAAYTDN VLDDFAYMTV LDVGSEVTFY GLEQYEKFPL ETGGSQRAAV VAAAAGISTA FATAWYLSQY

Mbo KVTLDVVAAG AALYDQIWLG SYMSGGVGFT QYATAAYTDN ILDDFTYMTV LDVGSEVTFY ALEQYDEYPL ETGGSQRAAV TAAASGCCTA FATAWYLSQY

Mar RSALDVVAAG AALYDQIWLG SYMSGGVGFT QYASAAYTDN ILDDFLYMTV LDVGSEVTFY GLEQYEEYPL ETGGSQRASV VSAAAGCATA FATAWYLSMY

Mfi KVTLDVVAQA AAIYDQIWLG SYMSGGVGFT QYASAAYTDN VLDDFAYMTV LDVGSEVTFY GLEQYEEYPL ETGGSQRASV VSAAAGISTA FATAWYLSMY

Mc1 RVSLDVVAQA AAIYDQIWLG SYMSGGVGFT QYASAAYTDN VLDDFCYMTV LDVGTEVTFY GLEQYEEYPL ETGGSQRASV VSAAAGISTA FATAWYLSMY

Mc2 RTTLDVVAMS AALYDQIWLG SYMSGGVGFT QYASAAYTDN VLDDFTYMTV LDVGSEVTFY GLEQYEEYPL ETGGSQRASV VSAAAGCSTA FATAWYLSMY

401 411 421 431 441 451 461 471 481 491 500

| | ***** | | | | | | | | |

Mor LHKEQHSRLG FYGFDLQDQC GAANVFSIRN DEGIPLEMRG PNYPNYAMNV GHQGEYAGIA QAPHSARGDA WAFNPLIKIA FADKNLIFDF SQPRAEFAKG

CS2 LHKEQHSRLG FYGFDLQDQC GAANVFSIRN DEGIPLEMRG PNYPNYAMNV GHQGEYAGIA QAPHSARGDA WAFNPLIKIA FADKNLIFDF SQPRAEFAKG

CS3 LHKEQHSRLG FYGFDLQDQC GAANVFSIRN DEGIPLEMRG PNYPNYAMNV GHQGEYAGIA QAPHSARGDA WAFNPLIKIA FADKNLIFDF SQPRAEFAKG

c86 LHKEQHSRLG FYGYDLQDQC GAANVFSIRN DEGLPLEMRG PNYPNYAMNV GHQGEYAGIA QAPHAARGDA FSFNPLVKIA FADKNLVFDF SKPRAEFAKG

ELS LHKEQHSRLG FYGYDLQDQC GAANVFSIRN DEGLPLEMRG PNYPNYAMNV GHQGEYAGIA QAPHAARGDA FSFNPLVKIA FADKNLVFDF SKPRAEFAKG

H10 LHKEQHSRLG FYGYDLQDQC GAANVFSIRN DEGLPLEMRG PNYPNYAMNV GHQGEYAGIA QAPHAARGDA FSFNPLVKIA FADKNLVFDF SKPRAEFAKG

c84 LHKEQHSRLG FYGFDLQDQC GAANVFSIRN DEGLPLEMRG PNYPNYAMNV GHQGEYAGIS QAPHAARGDA WAFNPLIKIA FADKNLAFDF SKVRSEFAKG

c94 LHKEQHSRLG FYGFDLQDQC GAANVFSIRN DEGLPLEMRG PNYPNYAMNV GHQGEYAGIS QAPHAARGDA WAFNPLIKIA FADKNLAFDF SKVRSEFAKG

B61 LHKEQHSRLG FYGFDLQDQC GAANVFSIRN DEGLPLEMRG PNYPNYAMNV GHQGEYAGIS QAPHAARGDA WAFNPLIKIA FADKNLAFDF SKVRSEFAKG

Mw1 LHKEQHSRLG FYGYDLQDQC GAANVFAIRN DEGLPLEMRG PNYPNYAMNV GHQGEYAGIA QAPHSARKDA WAFNPLIKIA FADKNLIFDF SKPRAEFAKG

Msm LHKEQHSRLG FYGYDLQDQC GAANVFSIRN DEGLPLEMRG PNYPNYAMNV GHQGEYAGIA QAPHAARGDA WSFNPLVKIA FADKNLVFDF SKPREEFAKG

Mmi LHKEQHSRLG FYGYDLQDQC GAANVFSIRN DEGLPLEMRG PNYPNYAMNV GHQGEYAGIA QAPHAARGDA WAFNPLIKIA FADKNLCFDF SQVRAEFAKG

Mth LHKEQHSRLG FYGFDLQDQC GAANVFSIRN DEGLPLEMRG PNYPNYAMNV GHQGEYAGIA QAPHSARGDA WAFNPLVKIA FADKNLVFDF SKVREEFAKG

Mgo LHKEQHSRLG FYGFDLQDQC GAANVFSIRN DEGLPLEMRG PNYPNYAMNV GHQGEYAGIA QAPHAARGDA FAFNPLIKIA FADKNLSFDF SKVRAEFAKG

Mol LHKEQHSRLG FYGFDLQDQC GAANVFAIRN DEGLPLELRG PNYPNYAMNV GHQGEYAGIA QAPHSARKDA YAVNPLVKIA FADKNLAFDF TKVRAEFAKG

Mru LHKEQHSRLG FYGFDLQDQC GAANVFAIRN DEGLPLELRG PNYPNYAMNV GHQGEYAGIA QAPHSARGDA FAVNPLVKIA FADKNLPFDF TKVRAEFAKG

Mw2 LHKEQHSRLG FYGYDLQDQC GAANTFSFRN DEGLPLEMRG PNYPNYAMNV GHQGEYAGIA QAPHAARGDA FTYNPLIKIA FADKNLVFDF SAPRNEIAKG

Mbo LHKEQHSRLG FYGYDLQDQC GAANVFSIRN DEGLPLEMRG PNYPNYAMNV GHQGEYAGIA QAPHAARKDA FSFNPLIKIA FADKNLCFDF TQPRAEIAKG

Mar LHKEQHSRLG FYGYDLQDQC GASNVFSIRN DEGLPVEMRG PNYPNYAMNV GHQGEYAGIS QAPHSARGDA FAFNPLVKIA FADQNLSFDF SQPRAEIAKG

Mfi LHKEQHSRLG FYGYDLQDQC GASNVFSIRN DEGLPVELRG PNYPNYAMNV GHQGEYTGIA QAPHAARGDA FAFNPLIKIA FADDNLSFDF SKPRAEIAKG

Mc1 LHKEQHSRLG FYGYDLQDQC GASNVFSIRN DEGLPVELRG PNYPNYAMNV GHQGEYSGIA QAPHAARKDA FTFNPLIKIA FADKNLSFDF SQPRAEIAKG

Mc2 LHKEQHSRLG FYGYDLQDQC GASNVFSIRA DEGLPVEMRG PNYPNYAMNV GHQGEYAGIS QAPHAARKDA FAFNPLIKIA FADKNLSFDF SQPRAEIAKG

Interactions in the catalytic site:

F430 axial ligant

CoB interaction

Post-translational modified

Part of the substrate cavity wall

CoM interaction

Asterisk indicates the only catalytic site with AA differences

M. oralis
TS-1
TS-2

**Figure S13.** Alignment of mcrA amino acid sequences from selected *Methanobrevibacter* genomes. Catalytic sites are indicated with color and an asterisk indicates the only site with an amino acid difference between the *Methanobrevibacter* spp.


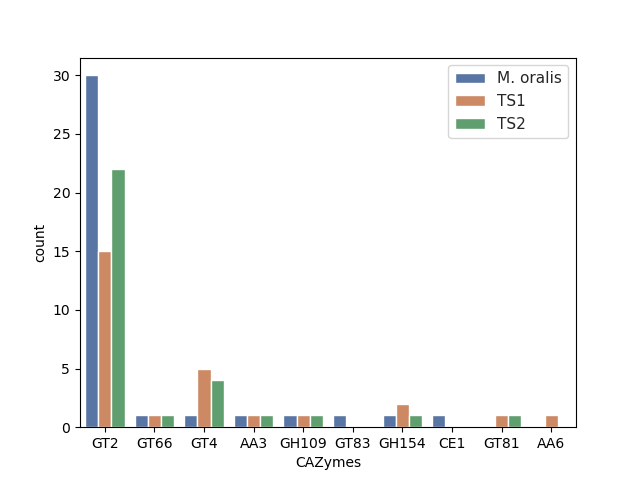


Figure S14. CAZyme counts in TS-1, TS-2 and *M. oralis*


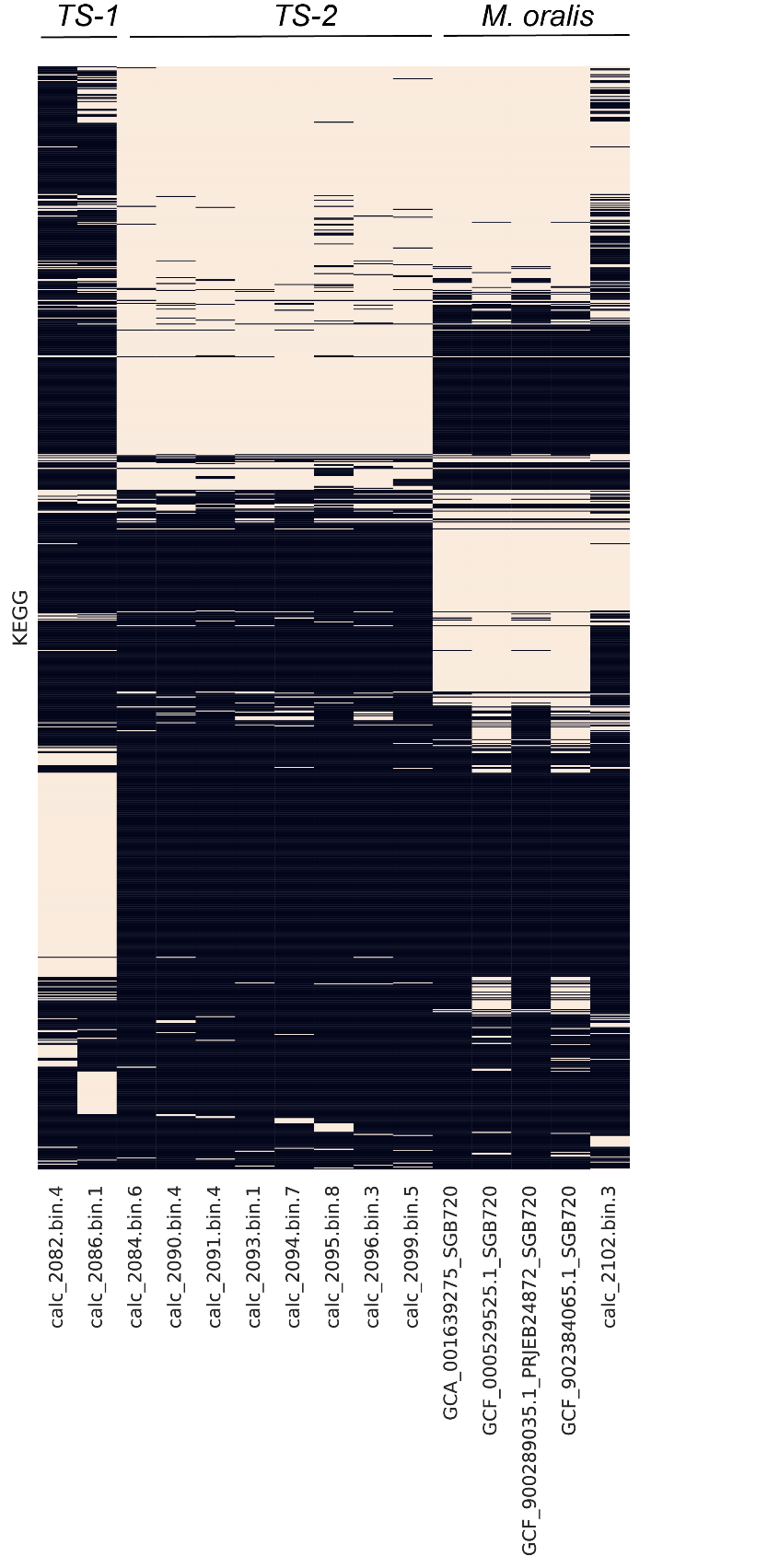


Figure S15. Absence and presence of genes (excluding those shared by all three species) identified with KEGG KO, across TS-1, TS-2 and *M. oralis* (light pink, present; black, absent)

**References**

1. Velsko, I.M., et al., *Microbial differences between dental plaque and historic dental calculus are related to oral biofilm maturation stage.* Microbiome, 2019. **7**(1): p. 102-102.

2. Mann, A.E., et al., *Differential preservation of endogenous human and microbial DNA in dental calculus and dentin.* Scientific reports, 2018. **8**(1): p. 9822-9822.

3. Ottoni, C., et al., *Metagenomic analysis of dental calculus in ancient Egyptian baboons.* Scientific Reports, 2019. **9**(1): p. 1-10.

4. Weyrich, L.S., et al., *Neanderthal behaviour, diet, and disease inferred from ancient DNA in dental calculus.* Nature, 2017. **544**(7650): p. 357-361.

5. Warinner, C., et al., *Pathogens and host immunity in the ancient human oral cavity.* Nature Genetics, 2014. **46**(4): p. 336-344.

6. Human Microbiome Project Consortium, *Structure, function and diversity of the healthy human microbiome.* Nature, 2012. **486**(7402): p. 207-14.

7. Johnston, E.R., et al., *Metagenomics Reveals Pervasive Bacterial Populations and Reduced Community Diversity across the Alaska Tundra Ecosystem.* Frontiers in Microbiology, 2016. **7**(579).

8. Festi, D., A. Putzer, and K. Oeggl, *Mid and late Holocene land-use changes in the Ötztal Alps, territory of the Neolithic Iceman “Ötzi”.* Quaternary International, 2014. **353**: p. 17-33.

9. Knights, D., et al., *Bayesian community-wide culture-independent microbial source tracking.* Nature Methods, 2011. **8**(9): p. 761-765.

10. Jónsson, H., et al., *mapDamage2.0: fast approximate Bayesian estimates of ancient DNA damage parameters.* Bioinformatics, 2013. **29**(13): p. 1682-1684.

11. Segata, N., et al., *Metagenomic biomarker discovery and explanation.* Genome Biology, 2011. **12**(6): p. R60.

12. Katoh, K. and D.M. Standley, *MAFFT multiple sequence alignment software version 7: improvements in performance and usability.* Mol. Biol. Evol., 2013. **30**(4): p. 772-780.

13. Didelot, X. and D.J. Wilson, *ClonalFrameML: Efficient Inference of Recombination in Whole Bacterial Genomes.* PLoS Comput. Biol., 2015. **11**(2): p. 1-18.
